# Targeting the epigenome and the integrated stress response to normalize colorectal cancer subclonal plasticity and progression

**DOI:** 10.1038/s41419-026-08720-2

**Published:** 2026-04-10

**Authors:** Lili Li, Taekyu Ha, Jing-Xin Feng, Michael DiPrima, Dunrui Wang, Parthav Jailwala, Thomas Meyer, Justin Lack, Hidetaka Ohnuki, Giovanna Tosato

**Affiliations:** 1 Laboratory of Cellular Oncology, Center for Cancer Research, National Cancer Institute (NCI), National Institutes of Health, Bethesda, MD USA; 2 Advanced Biomedical Computational Science, Frederick National Laboratory for Cancer Research, Frederick, MD USA

**Keywords:** Cancer, Cancer genetics

## Abstract

Despite therapeutic advances, metastatic colorectal cancer remains a therapeutic challenge as most patients will develop resistance to therapy and will progress. Epigenetic mechanisms are implicated in enabling the acquisition of new phenotypic traits as drivers of colorectal cancer progression, rather than new genetic mutations or expansion of existing mutant clones. It remains unclear, however, which epigenetic mechanisms sustain colorectal cancer plasticity, how they are induced, and how this plasticity generates subclonal diversity that drives the aggressive cancer phenotype. Here we identify the integrated stress response as an inducer of colorectal cancer cell plasticity, subclonal diversity, and tumor progression in the stress-surviving cells. Combined analysis of chromatin accessibility and gene transcription profiling in these cells found the emergence of an endogenous interferon response as a key phenotypic trait associated with subclonal colorectal cancer cell diversity, treatment resistance and heightened aggressiveness. We unveil a new experimental approach to successfully prevent treatment-resistant colorectal cancer progression by combining epigenetic modulators with a cereblon-dependent degrader of GSPT1, a regulator of protein synthesis, to normalize chromatin accessibility and induce colorectal cancer cell death. Collectively, our study identifies the integrated stress response as an inducer of epigenetic and transcriptional plasticity in colorectal cancer cells and highlights a successful approach to therapeutic intervention.

## Introduction

Colorectal cancer (CRC) is the third most common cancer worldwide and the second leading cause of all cancer-related deaths (International Agency for Research on Cancer, IARC). Treatment of CRC has evolved with the introduction of targeted therapies against VEGFR, EGFR and immunotherapy supplementing chemotherapy, resulting in improved patient survival [1–3]. However, metastatic disease, which eventually develops in about 50% of all CRC patients, is a therapeutic challenge, with a 5-year survival of less than 15% [4].

Phylogenetic analyses of bulk DNA from primary and metastatic CRC have shown that primary and metastatic CRC share the same types and number of genetic mutations, with little genomic divergence; they also share similar chromosomal instability status [5–7]. Furthermore, progressing CRC infrequently shows expansions of the genetically mutant clones originally present [8–10]. These observations support a model where most genetic driver events are acquired early during cancer evolution and progressive acquisition of mutations and clonal expansions are not cause of metastatic CRC disease [10–12].

Recent studies have highlighted a critical role for cell “plasticity”, independent of genetic traits, as a contributor to CRC heterogeneity and tumor evolution, and suggested that additional layers of regulation, particularly epigenetic alterations, drive the plasticity and phenotypic changes required for CRC evolution and metastasis [12–14]. However, current understanding of the mechanisms underlying CRC plasticity, how they contribute to CRC progression and aggressiveness remains limited, potentially preventing identification of new therapeutic opportunities.

In this study, we uncovered the integrated stress response (ISR) [15, 16] as a key inducer of altered chromatin accessibility and subversion of gene transcription that drive subclonal CRC aggressiveness and we outline a successful experimental approach to reverse the resistant phenotype based on this new understanding.

## Results

### Emergence of a resistant subclonal population of CRC cells

We previously introduced a doxycycline (Dox)-inducible shRNA (#596) to deplete the pro-survival Ephrin B2 protein [17, 18] from the human HT29 colon cancer cell line, and selected a single clone of HT29 cells, named 596-7, where the induced shRNA expression is lethal for most cells [19]. The clonality of 596-7 cells was confirmed genetically, as 10/10 subclones of 596-7 displayed the same 15 shRNA integration sites [19].

**Fig. 1:**
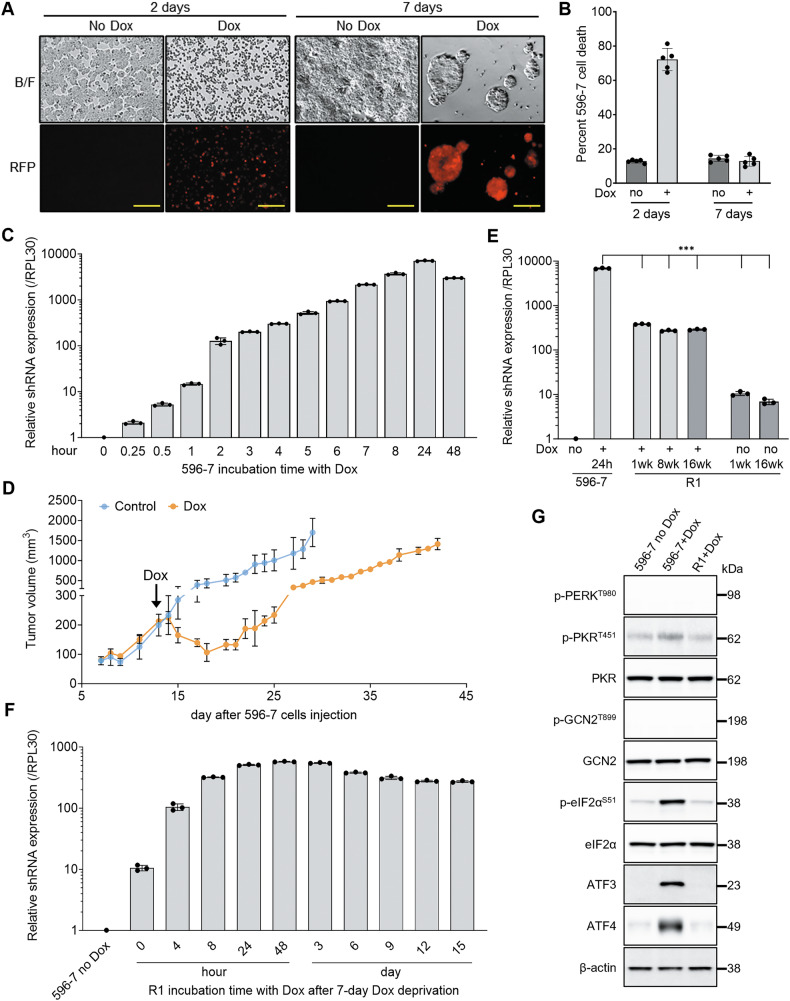
Emergence of viable 596-7 cells after undergoing a stress response from activation of the inducible integrated shRNA. **A** Representative bright-field and immunofluorescence (RFP) images of 596-7 cells cultured 2 or 7 days with or without Doxycycline (Dox) to induce expression of shRNA596. Most cells appear dead 2 days after exposure to Dox. Live cell clusters emerge 7 days after culture with Dox. The RFP fluorescence indicates expression of the Dox-inducible shRNA. Scale bar: 200 μm. **B** Percent 596-7 cell death after 2- and 7-day culture with or without Dox by acridine orange/propidium iodide staining; *n* = 5 experiments; error bars ± standard deviation (SD). **C** Time course of Dox-induced shRNA expression in 596-7 cells. Results of qPCR are normalized by RPL30 mRNA and expressed as relative to the expression in 596-7 cells maintained without Dox. The results reflect triplicate measurements; each dot represents one of the replicate measurements. Representative experiment of 3 performed. **D** Emergence of tumors from s.c. inoculation of 596-7 cells infected with shRNA596 into NOD/SCID mice (10 mice/group). One group of mice received Dox chow beginning on day 13 after cell injection, 6 days after the tumors emerged (pointed by the arrow); the other group received regular chow. The results reflect the mean (± SD) tumor volume/group. **E** Relative shRNA expression by qPCR. 596-7 cells cultured with or without Dox for 24 h (h); R1 was cultured with Dox from emergence for 1, 8 or 16 weeks (wk), or was deprived of Dox for 1 or 16 wk, after culture with Dox for 16 wk. Results, normalized by RPL30 mRNA, are expressed as relative to the expression in 596-7 cells maintained without Dox. The results reflect triplicate measurements; each dot represents one of the replicate measurements. Representative of 3 experiments performed. ***P < 0.001 by unpaired Student’s t-test compared to 596-7 cells + Dox (24 h). **F** R1 was deprived of Dox for 7 days and then re-exposed to Dox for the indicated time intervals. Results of qPCR normalized by RPL30 mRNA are expressed as relative to the expression in 596-7 cells maintained without Dox. The results reflect triplicate measurements; each dot represents one of the replicate measurements. Representative experiment of 3 performed. **G** ISR markers, detected in 596-7 cells treated with Dox (8 h), are not detected in R1 (incubated continuously with Dox for 4 months since emerging from Dox-treated 596-7) and in 596-7 cells never exposed to Dox. Representative immunoblotting results from one of three experiments.

We now confirmed that clonal 596-7 cells reproducibly die two days after addition of Dox, associated with substantial activation of the shRNA (~8000-fold induction) and reporter-derived red fluorescent protein (RFP) (Fig. 1A–C and Supplementary Fig. S1A). Additionally, we observed the emergence of viable 596-7 cell colonies by day 7, despite the continuous presence of Dox, establishing a sub-clonal cell line, designated “R1” (resistant 1; Fig. 1A). Emergence of R1 from Dox-induced 596-7 cells was recapitulated in NOD/SCID mice bearing tumor xenografts from inoculation of the 596-7 cells (containing sh596). The mice treated with Dox-containing chow initially showed a reduction in tumor size, followed by regrowth; control mice showed progressively growing tumors (Fig. 1D).

R1 emerging from Dox-activated 596-7 cells and after prolonged culture (1-16 weeks with Dox) proliferated less than 596-7 cells never exposed to Dox (596-7 no Dox) but were similarly viable (Supplementary Fig. S1A, B). Removal of Dox (1 or 16 weeks) from R1 previously cultured with Dox did not change R1 proliferation (Supplementary Fig. S1B).

Previously, we determined that death of Dox-treated 596-7 cells is driven by activation of the “integrated stress response” (ISR) initiated by the protein kinase RNA-induced (PKR) responding to excessive shRNA expression [19]. The ISR, an evolutionary conserved pathway that regulates cell adaptation to various stressors, leads to cell death, eliminating damaged cells, or to cell survival, in part dependent upon stress intensity [15, 16]. We now found that expression of the integrated shRNA in R1 when it emerges from Dox-activated 596-7 cells, or after culture (1-16 weeks) with Dox is significantly reduced (~10-fold) compared to 596-7 cells initially Dox-induced (24 h; when ISR is present) (Fig. 1E). Also, we found that Dox-deprived (1 or 16 weeks) R1 after extended culture with Dox, maintains expression of the integrated shRNA, albeit at lower levels than in R1 maintained with Dox (Fig. 1E). Re-introduction of Dox to Dox-deprived (1 week) R1 induces re-expression of the integrated shRNA with similar kinetics but at lower levels (~10-fold lower) than in 596-7 first induced with Dox (Fig. 1F). These differences between R1 compared to 596-7 cells were not attributable to genetic changes in the shRNA^miR^ sequence (Supplementary Fig. S1C).

The ISR markers p-eIF2α, ATF3 and ATF4 were not detected in Dox-maintained R1 that expresses the integrated shRNA at lower levels than in Dox-induced (24 h) 596-7 cells (Fig. 1G), leading us to hypothesize that emergence of R1 from Dox-activated 596-7 cells reflects R1 failure to express the integrated shRNA at sufficiently high levels to induce ISR.

### Transcriptional reprogramming and interferon pathway activation in R1

**Fig. 2:**
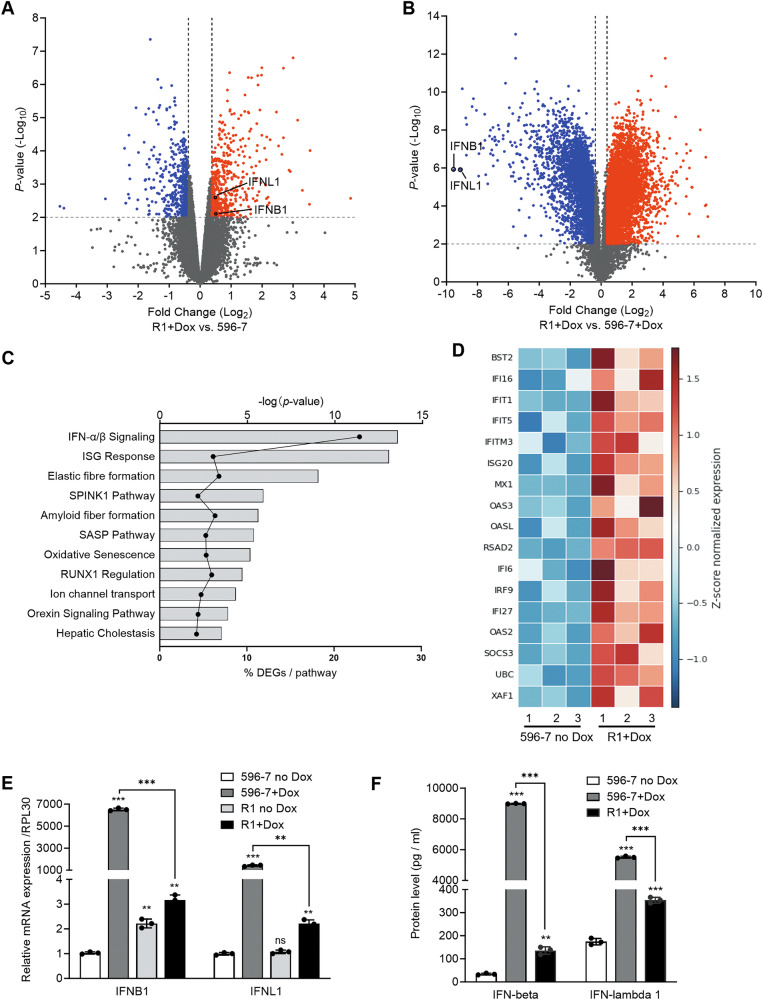
Comparative transcriptome analysis of R1 and 596-7 cells. A,B Volcano plots showing the magnitude (fold change) and statistical significance of changes in RNA expression between **A** R1 propagated in medium with Dox (*n* = 3; R1+Dox) versus 596-7 cells (*n* = 3; never exposed to Dox); and **B** between R1+Dox cells versus 596-7 cells exposed to Dox for 24 h (*n* = 4; 596-7+Dox). Each dot in the volcano plot represents an annotated RNA measured by RNA-seq. Significantly (*P* < 0.01; fold change >1.3) induced RNAs are shown as red dots and significantly repressed RNAs are represented by blue dots. Vertical and horizontal lines reflect cutoffs for fold change and significance. *IFNB1* and *IFNL1* expression changes are indicated. **C** Canonical pathway enrichment analysis (Ingenuity Pathway Analysis) showing differences between R1+Dox (R1 propagated with Dox) vs 596-7 cells (never exposed to Dox). Input data are all genes differentially expressed (*P*-value < 0.05, fold change >1.3) in the two groups. The bar graph identifies all the IPA pathways showing a *P*-value (<0.01) and a Z-score (≥2), ordered by % differentially expressed genes (DEGs) in the pathway/total number of genes that map to that pathway. The black dots on each pathway bar correspond to the -log of the P value (Fisher’s exact test). **D** Heatmap showing selected IFN-pathway-related genes expressed at significantly (P < 0.01) higher levels in R1+ Dox (R1 propagated with Dox) compared to 596-7 cells no Dox (never Dox) by row-wide Z-score (color bar). These 17 genes were identified by the “interferon signaling pathway” and “negative regulation of viral genome replication” pathway. The numbers 1, 2, and 3 indicate RNA-seq samples. **E**, **F** Relative *IFNB1* and *IFNL1* mRNA expression by qPCR (**E**) and IFN beta and lambda 1 protein levels (**F**) in 596-7 cells (never Dox); 596-7 cells +Dox (24 hours); in R1 no Dox (cultured without Dox for 4 months after been maintained in Dox for 4 months), and R1+Dox (cultured with Dox for 4 months). Results in (**E**) are presented as mean (± SD) relative values (normalized by RPL30 mRNA expression); representative of 3 experiments; each dot represents one of the replicate measurements; results in F are presented as mean (± SD; triplicate samples) protein levels (pg/ml); representative of 3 experiments; ns, not significant; **P < 0.01, ***P < 0.001 by unpaired Student’s t-test compared to 596-7 no Dox (unless otherwise noted).

We compared the transcriptome of R1 (maintained in Dox; R1+Dox) to that of 596-7 never exposed to Dox or Dox-induced for 24 h (Fig. 2A, B). RNA-seq identified 925 differentially expressed transcripts (*P* < 0.01, fold change >1.3) in R1+Dox compared to 596-7 cells never Dox (Fig. 2A and Supplementary Fig. S2A), and 8 178 differentially expressed transcripts (*P* < 0.01, fold change >1.3) in R1+Dox compared to Dox-induced 596-7 cells (Fig. 2B).

We queried Ingenuity Pathway Analysis (IPA). Among 516 IPA “canonical pathways”, only 11 met the criteria of significance (*P* value < 0.01) and Z-score (≥ 2, indicative of activity) distinguishing R1+Dox from 596 cells never Dox (Fig. 2C). Of these, “Interferon alpha/beta signaling” was predicted to be most significantly active in R1 compared to 596-7 cells (Fig. 2C). Additionally, Gene Ontology (GO; Biological Process Enrichment) identified “negative regulation of viral genome replication” as the pathway populated by the largest proportion of differentially expressed genes (DEGs) in R1 compared to 596-7 cells (Supplementary Fig. S2B). Thus, “Interferon alpha/beta signaling” and “negative regulation of viral genome replication” identified 17 genes expressed significantly more in R1 compared to 596-7 cells (Fig. 2D).

Consistently, R1+Dox expressed more *IFNB1* and *IFNL1* mRNA and interferon (IFN)-beta and IFN-lambda 1 proteins than 596-7 cells never-Dox, but these levels in R1+Dox are significantly lower than in 596-7 cells+Dox (24 h) (Fig. 2E, F). These results suggested that R1+Dox retains an active interferon response restraining expression of the integrated shRNA.

Whole-genome sequencing demonstrated that R1 retains all the canonical driver mutations present in the parental HT29 line (APC, TP53, BRAF, PIK3CA, and SMAD4) and RNA-seq showed comparable expression levels of these driver genes in R1 and 596-7 cells (Supplementary Fig. S2C, D). The overall mutational burden was similar in R1 and 596-7 (935 vs 942 mutations), the majority of which were shared (595 common variants) (Supplementary Fig. S2E). Only 59 variants were unique to R1, and of these, 18 somatic mutations were confirmed at sequencing depth ≥10x, comprising 11 coding sequence changes and 7 splice-region alterations (Supplementary Fig. S2F), but none of these are known or predicted contributors to cancer growth or drug resistance. Thus, the outgrowth of R1 from Dox-induced 596-7 cells is unlikely driven by R1 acquisition of new oncogenic mutations, suggesting instead development of epigenetic reprogramming.

### Defective shRNA promoter activation in R1

**Fig. 3:**
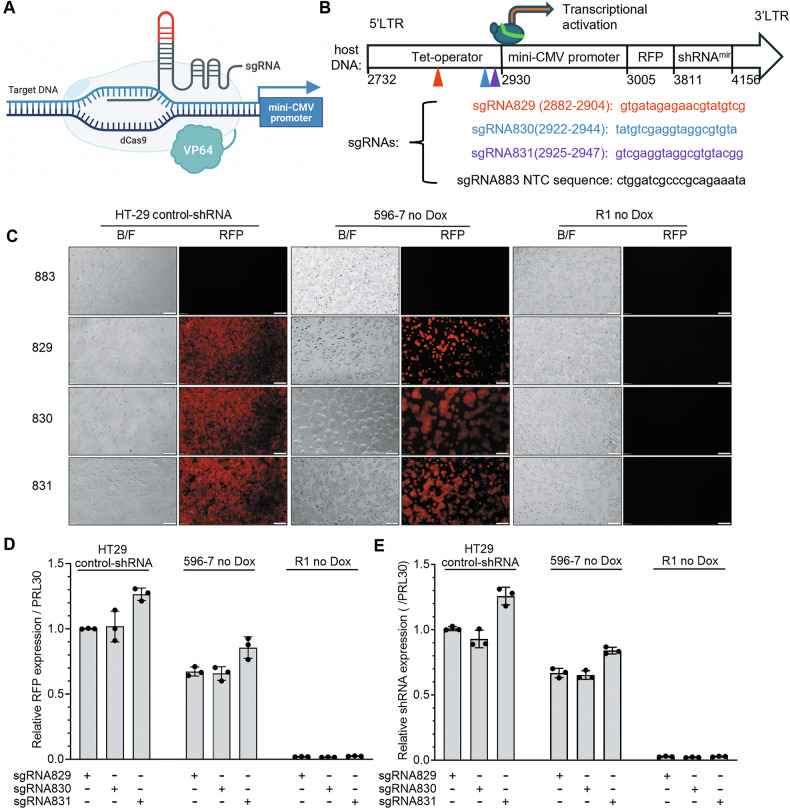
shRNA expression and processing in R1. **A** Schematic representation of the dCas9-sgRNA system designed to promote activation of the mini-CMV promoter in the pTRIPZ-CMV vector. sgRNAs are designed to recognize and bind to a target DNA sequence upstream of the mini-CMV promoter. The catalytically inactive Cas9 (dCas9) fused to the VP64 transcriptional activation domain forms a complex with the sgRNA. The dCas9-VP64/sgRNA complex binds to the target DNA sequence to activate the mini-CMV promoter and drive shRNA expression. **B** The mini-CMV promoter that drives RFP and shRNA expression in the pTRIPZ-CMV-shRNA^miR^ vector is shown schematically. The numbers below the vector indicate bp number. sgRNA sequences and their targeting sites on the pTRIPZ-CMV-shRNA^miR^ plasmid are shown. The sgRNAs include a non-Mammalian targeting (NTC) control (#883) and the mini-CMV promoter targeting sgRNAs #829, #830 and #831. **C** The targeting sgRNAs (829, 830, 831) induce visible RFP fluorescence in HT29 infected with the control-shRNA (non-Mammalian shRNA control SHC002) and in 596-7 no Dox (never Dox), but not in R1 no Dox (without Dox 4 months). Control sgRNA883 (non-targeting) induced no visible RFP fluorescence. Representative images from bright field (BF) and red fluorescence protein (RFP) imaging through an Olympus microscope. Representative images from one of three experiments. **D**, **E** Expression levels of RFP (**D**) and shRNA (**E**) in HT29 (infected with control-shRNA), 596-7 cells (infected with sh596), and R1 (derived from 596-7 cells) after induction with the targeting sgRNAs (829, 830, 831). Results from qPCR are expressed as relative to the expression of HT29 control-shRNA infected with sgRNA829. The results reflect the means (± SD) of triplicate measurements; each dot represents one of the replicate measurements. Representative results from 3 experiments.

We tested if epigenetic reprogramming limits R1 from fully activating the integrated shRNA596 by targeting the shRNA promoter with a CRISPR-based transcriptional activator. We used the dCas9-VP64 system with three sgRNAs (#829, #830, #831) designed to bind to different sites of the mini-CMV promoter, along with a non-targeting sgRNA control (#883) (Fig. 3A, B) to compare sgRNA-induced activation of shRNA596 in R1 and 596-7 cells. As a control, we included HT29 cells infected with the non-mammalian pTRIPZ-CMV-shRNA^mir^ vector only, the same vector present in 596-7 and R1 (Supplementary Fig. S3A). This experiment shows that all the promoter-targeting sgRNAs (#829, #830, #831) induced minimal RFP fluorescence and shRNA expression in R1 but induced robust RFP fluorescence and shRNA expression in HT29 and 596-7 cells; the non-targeting control sgRNA (#883) had no effect (Fig. 3C–E). This failure of R1 to activate the integrated shRNA could not be attributed to R1 expressing lower levels of dCas9 and sgRNAs than 596-7 and HT29 cells (Supplementary Fig. S3B–D). Together, these results show that R1 is refractory to promoter-targeted reactivation of the integrated shRNA, suggesting impaired chromatin accessibility in the shRNA.

### R1 displays altered chromatin accessibility

**Fig. 4:**
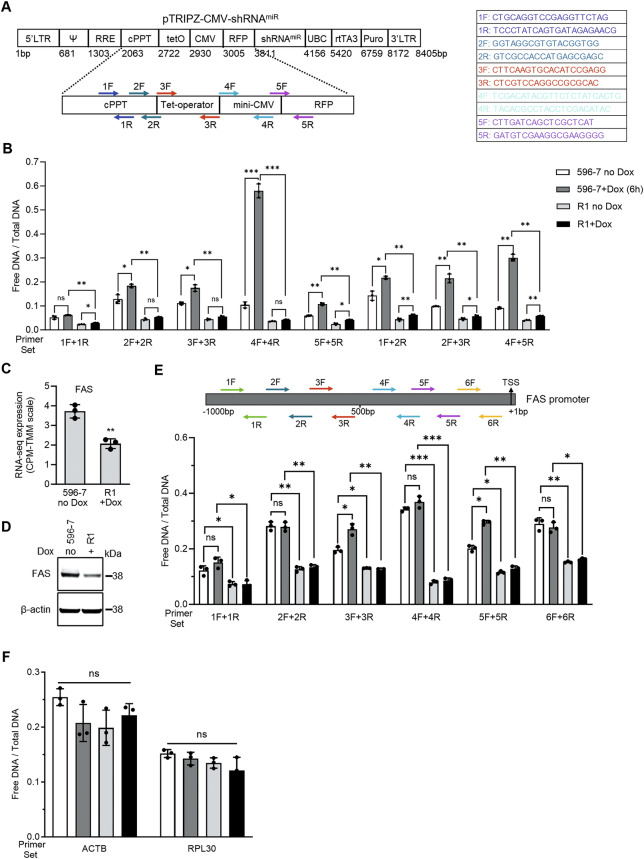
Accessibility of chromatin in the shRNA596 and FAS promoters. **A** Schematic representation of primer pairs designed to amplify 60-150 bp DNA fragments within the pTRIPZ-CMV-shRNA^miR^ sequences upstream of the RFP sequence. The 5 pairs of primer sequences are shown on the right. **B** FAIRE (Formaldehyde-Assisted Isolation of Regulatory Elements) followed by quantitative analysis by PCR (FAIRE-qPCR) was used to characterize chromatin accessibility in the integrated shRNA596 in 596-7 cells exposed (6 h) or never exposed to Dox; R1 deprived of Dox for 4 months (R1 no Dox); and R1 maintained in Dox for 4 months (R1+Dox). The results are expressed as a ratio of free DNA/total input DNA ( ± SD; triplicate measurements), each point represents one replicate. Representative of 3 experiments. **C**, **D** *FAS* mRNA by RNA-seq (**C**) and FAS protein by western blotting (**D**) in 596-7 cells never exposed to Dox and R1 propagated with Dox. The results of RNA-seq from triplicate samples are presented as means (± SD); western blot results are representative of 3 experiments. **E** Schematic of *FAS* promoter and primer sets used for FAIRE-qPCR analysis (top). The bar graph shows the results of FAIRE-qPCR analysis in 596-7 exposed (6 hours) or never exposed to Dox; R1 deprived of Dox for 4 months (R1 no Dox); and R1 maintained in Dox for 4 months (R1+Dox). The results are expressed as a ratio of free DNA/total input DNA ( ± SD; triplicate measurements). Representative of 3 experiments. **F** Accessibility of chromatin in the *ACTB* and *RPL30* promoters of by FAIRE-qPCR in 596-7 exposed (6 h) or never exposed to Dox; R1 deprived of Dox for 4 months (R1 no Dox); and R1 maintained in Dox for 4 months (R1+Dox). The results are expressed as a ratio of free DNA/total input DNA ( ± SD; triplicate measurements); representative of 3 experiments. In this figure, ns not significant; *P < 0.05, **P < 0.01, ***P < 0.001 by unpaired Student’s t-test compared to 596-7 no Dox.

We assessed the chromatin status of the mini-CMV promoter that drives expression of the integrated shRNA by Formaldehyde-Assisted Isolation of Regulatory Elements followed by qPCR (FAIRE-qPCR) [20], using 5 sets of closely spaced primers designed to amplify (60–150 bp) in and around the region of interest (Fig. 4A). The results show that R1 (propagated with or without Dox) displays a reduced chromatin accessibility in the region of interest compared to 596-7 never-Dox or Dox-exposed (6 h+Dox) (Fig. 4B).

Since the opening of chromatin is an important prerequisite for DNA transcription [21], and RNA-seq indicated extensive changes in gene expression in R1 compared to 596-7 cells, we hypothesized that R1 has acquired broad changes in chromatin accessibility. We examined the *FAS* promoter because *FAS* mRNA and protein levels are reduced in R1 compared to 596-7 cells (Fig. 4C, D). FAIRE-qPCR results showed reduced chromatin accessibility in the *FAS* promoter of R1 compared to 596-7 cells (Fig. 4E). Control FAIRE-qPCR results detected similar chromatin accessibility in the *ACTB* and *RPL30* promoters of R1 (no Dox or +Dox) and 596-7 cells (no Dox or +Dox), supporting the specificity of reduced chromatin accessibility at the mini-CMV and *FAS* promoters (Fig. 4F).

Consistently, bisulfite sequencing detected four CpG sites within 800 bp from the *FAS* transcription start site with increased methylation in R1+Dox compared to 596-7 cells no Dox (Supplementary Fig. S4A, B). Additionally, Chromatin Immunoprecipitation sequencing (ChIP-seq) of the *FAS* promoter detected increased tri-methylation (3me) of the transcriptional repressive marks histone 3 (H3) lysine-9 (K9) (H3K9me3) and H3K27me3, and decreased tri-methylation of the transcriptional activator H3K4me3 in R1 compared 596-7 cells (Supplementary Fig. S4C), supporting functional impairment of the *FAS* promoter.

We also compared chromatin accessibility in the *IFNB1* and *IFNL1* promoters since *IFNB1* and *IFNL1* mRNA and protein levels are increased in R1+Dox compared to 596-7 cells no Dox (Fig. 2E, F). This increase, however, is small compared to levels of *IFNB1* and *IFNL1* mRNA and protein induced by Dox in 596-7 cells (Fig. 2E, F). FAIRE-qPCR showed increased chromatin accessibility in the *IFNB1* and *IFNL1* promoters in R1 compared to 596-7 cells (Supplementary Fig. S4D, E), but decreased chromatin accessibility in the *IFNB1* and *IFNL1* promoters in R1 compared to Dox-induced 596-7 cells (Supplementary Fig. S4D, E).

Overall, these results suggested that R1 has undergone complex chromatin remodeling. Supporting this possibility, interferon signaling, more active in R1 compared to 596-7 cells, broadly reshapes chromatin by producing both increased and decreased accessibility at interferon-target genes, changes critical for IFN-induced transcriptional control [22, 23].

### Effects of epigenetic drugs on chromatin accessibility and gene expression in R1

If R1 displays broadly altered chromatin accessibility compared to 596-7 cells, and these changes are key to the death-resistant phenotype of R1, epigenetic drugs could reverse this phenotype. To test this possibility, we selected the DNA methyltransferase inhibitor 5-Aza-2’-Deoxycytidine (5 AZaD) that causes DNA demethylation [24–26] and the histone methyltransferase inhibitor 3-Deazaneplanocin A (DZNep), a global inhibitor of histone methylation [27].

**Fig. 5:**
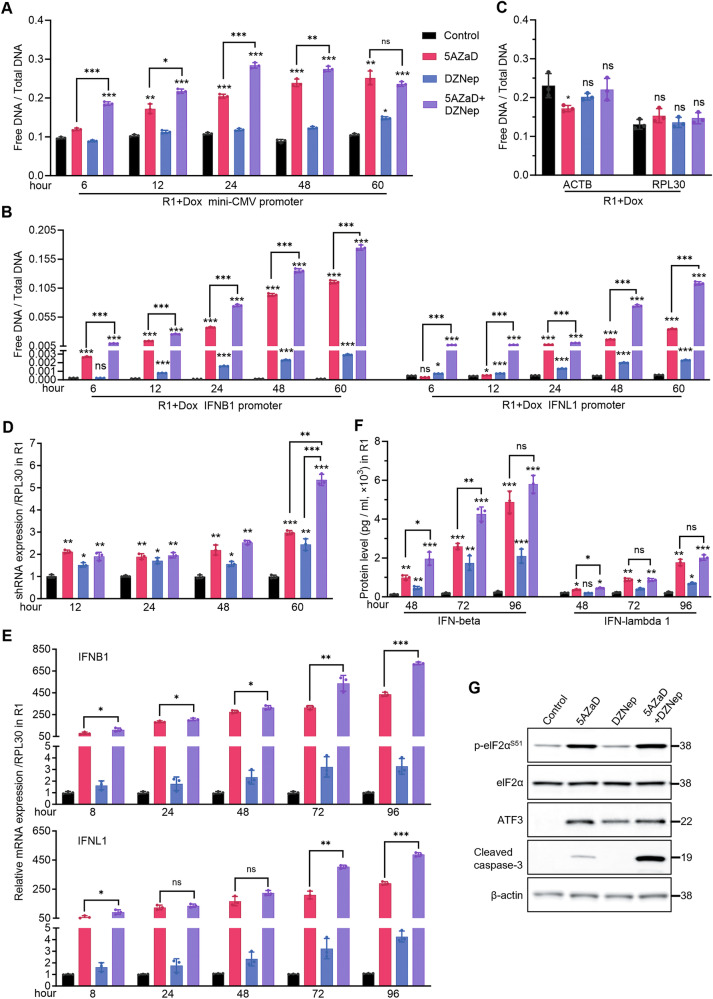
The drugs 5-AzaD and DZNep regulate chromatin accessibility and gene expression in R1. **A**,**B** FAIRE-qPCR analysis of accessibility to chromatin at the shRNA mini-CMV (**A**), *IFNB1* and *IFNL1* (**B**) promoters in R1 maintained in Dox (R1+Dox), after 6–60 h (h) incubation in medium with 0.1% DMSO, with 5AZaD, with DZNep, or with the combination of 5AZaD+DZNep. The results are expressed as a ratio of free DNA/total input DNA (mean ± SD; triplicate measurements), each point represents one replicate. Representative of 3 experiments. **C** FAIRE-qPCR analysis of chromatin accessibility at the *ACTB* and *RPL30* promoters in R1 maintained in Dox (R1+Dox) after 60 h incubation with medium with 0.1% DMSO, with 5AZaD, with DZNep, or with the combination of 5AZaD+DZNep. The results are expressed as a ratio of free DNA/total input DNA (mean ± SD; triplicate measurements), each point represents one replicate. Representative of 3 experiments. **D** qPCR analysis of shRNA expression in R1+Dox cells incubated 12–60 h (h) in medium with 0.1% DMSO, with 5AZaD, with DZNep, or with the combination of 5AZaD+DZNep (as in **A**). Expression values, normalized by *RPL30*, are displayed as relative to expression of R1 in medium with 0.1% DMSO only; mean (± SD of triplicate measurements). Representative of 3 experiments. **E** qPCR analysis of *IFNB1* (top) and *IFNL1* (bottom) mRNA expression in R1+Dox cells treated as in (**A**) for 8 to 96 h (h). Expression values are presented as relative values and reflect the mean (± SD of triplicate measurements). Representative of 3 experiments. **F** ELISA quantification of secreted IFN-beta and IFN-lambda 1 in supernatants of R1+Dox cells after 48–96 h incubation in medium only (with 0.1% DMSO), with 5AZaD, with DZNep or with 5AZaD+DZNep (as in **A**). The results reflect the mean (± SD of triplicate measurements). Representative of 2 experiments. **G**. Western blot analysis of the ISR marker proteins p-eIF2α^S51^ and ATF3, and the cell death marker Cleaved caspase-3. R1+Dox were incubated (72 h) in medium only (with 0.1% DMSO), with 5AZaD, with DZNep, or with 5AZaD+DZNep (as in A). Representative of three experiments. Throughout, cells were cultured in medium only (includes 0.1% DMSO), with 5AZaD (500 nM), with DZNep (200 nM), or with the combination of 5AZaD (500 nM) plus DZNep (200 nM). Error bars represent the mean (± SD) from triplicate measurements; *P* values from unpaired Student’s t-test are calculated relative to the untreated control; ns: not significant; *P < 0.05, **P < 0.01, ***P < 0.001.

In R1+Dox, 5 AZaD time-dependently increased chromatin accessibility at the shRNA mini-CMV promoter (Fig. 5A) and the *IFNB* and *IFNL* promoters (Fig. 5B), whereas DZNep alone was less effective. Together, DZNep+5AZaD enhanced significantly chromatin accessibility at these sites (Fig. 5A, B). Control experiments showed that 5AZaD and DZNep, individually or together, minimally changed chromatin accessibility at the *ACTB* and *RPL30* promoters in R1+Dox and in 596-7 cells with (8 hours; 596-7+Dox) or without Dox (Fig. 5C and Supplementary Fig. S5A).

Consistently, 5AZaD+DZNep increased (*p* < 0.001; 60 h time-point) shRNA expression in R1+Dox (Fig. 5D), raising shRNA expression levels to 49.3% of peak shRNA expression in 596-7+Dox cells, and less so in R1 no Dox (Supplementary Fig. S5B). Also, 5AZaD+DZNep increased significantly expression of *IFNB1, IFNL1* and *FAS* (Fig. 5E and Supplementary Fig. S5C) in R1+Dox, raising *IFNB1*, *IFNL1* and *FAS* expression to 72.8%, 61.1% and 112.8%, respectively, of those achieved in 596-7+Dox cells (Supplementary Fig. S5B). Similarly, IFN-beta and IFN-lambda 1 levels were increased in culture supernatants of R1 incubated with 5AZaD+DZNep (Fig. 5F). Additionally, 5AZaD and DZNep induced ISR activation associated with the cell-death marker cleaved caspase-3 in R1+Dox (Fig. 5G). Together, these results indicate that 5AZaD+DZNep remodel chromatin; promote expression of the integrated shRNA and the *IFNB1*, *IFNL1*, and *FAS* genes; and activate the ISR in R1.

### 5AZaD and DZNep reduce R1 viability, proliferation, and tumor growth

**Fig. 6:**
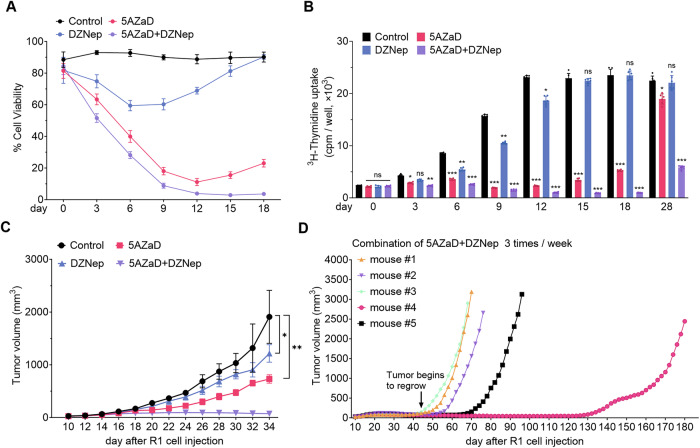
Epigenetic drug treatment reduces the growth and promotes cell death in R1. **A** Cell viability of R1+Dox (maintained in Dox) cultured with 5AZaD (500 nM), DZNep (200 nM), or 5AZaD (500 nM) + DZNep (200 nM) assessed by acridine orange/propidium iodide staining at the indicated times (0–18 days). Control cells were incubated in culture medium containing 0.1% DMSO. The results reflect the means from triplicate measurements (SD). Representative of 3 experiments. **B** Cell proliferation measured by ^3^H-thymidine incorporation in R1+Dox incubated 0 to 28 days in medium only (contains 0.1% DMSO) or with 5AZaD, DZNep, or 5AZaD+DZNep as in (**A**). Error bars represent the mean ± SD from six replicates; each point represents one replicate; *p* values from unpaired Student’s t-test are calculated relative to the untreated control; ns: not significant; *P < 0.05, **P < 0.01, ***P < 0.001. **C** Effects of 5AZaD, DZNep and 5AZaD+DZNep on R1 tumor growth in NOD/SCID mice (*n* = 5 per group). Mice were injected s.c. with R1+Dox cells and fed doxycycline chow. When tumors became measurable (day 10), mice were treated intraperitoneally (i.p.) with 5AZaD (1 mg/kg), DZNep (0.5 mg/kg), 5AZaD (1 mg/kg) + DZNep (0.5 mg/kg), or diluent only (Control), three times per week. Tumor volume is displayed as mean (± SD) at the indicated time-points. **D** Extended observation of NOD/SCID mice bearing R1 tumors and treated with 5AZaD+DZNep (shown in (**C**), up to day 34); tumor regrowth occurred in each of the 5 mice, albeit at significantly different times. Individual tumor growth curves of R1+Dox xenografts in NOD/SCID mice treated with the combination of 5AZaD and DZNep.

5AZaD+DZNep induced a progressive loss of cell viability in R1 cultures; by day 12, only <10% of cells were alive. Individually, DZNep and 5AZaD only transiently reduced R1 viability (Fig. 6A and Supplementary Fig. S6A). Consistently, 5AZaD+DZNep reduced R1+Dox proliferation up to day 18. However, by day 28 R1+Dox had resumed proliferation despite 5AZaD+DZNep (Fig. 6B). In R1 no Dox, 5AZaD+DZNep reduced the proliferation less than in R1+Dox cells (Supplementary Fig. S6B).

We tested the effects of 5AZaD and DZNep on R1 tumor growth. NOD/SCID mice inoculated with R1+Dox cells were fed Dox-containing chow. When tumors became measurable, mice were randomized to receive diluent only (Control), 5AZaD (1 mg/kg, i.p.), DZNep (0.5 mg/kg i.p.) or the combination of 5AZaD (1 mg/kg) + DZNep (0.5 mg/kg, i.p.). Drug concentrations were selected from dose-finding toxicity studies [27, 28]. 5AZaD+DZNep blocked R1 tumor growth over 34 days from cell inoculation, whereas individually, 5AZaD and DZNep were less effective (Fig. 6C).

We continued 5AZaD+DZNep treatment in the 5 mice with no tumor growth on day 34. By day 45, 2/5 tumors had begun to regrow, and by day 72, 4/5 tumors had regrown; the remaining tumor began to regrow by day 122 (Fig. 6D). Overall, these results indicate that 5AzaD+DZNep effectively reduce R1 proliferation in vitro and R1 tumor growth.

### Activity of 5AZaD and DZNep combined with the GSPT1 degrader, CC-90009

Since 5AZaD+DZNep did not cure mice of R1 tumors and ISR activation is a key inducer of cell death in Dox-treated 596-7 cells, we sought to amplify the ISR induced by 5AZaD plus DZNep (Fig. 5G). CC-90009 is an immunomodulatory imide drug that activates the ISR in tumor cells through cereblon-dependent degradation of GSPT1, which regulates translation termination [29–31].

**Fig. 7:**
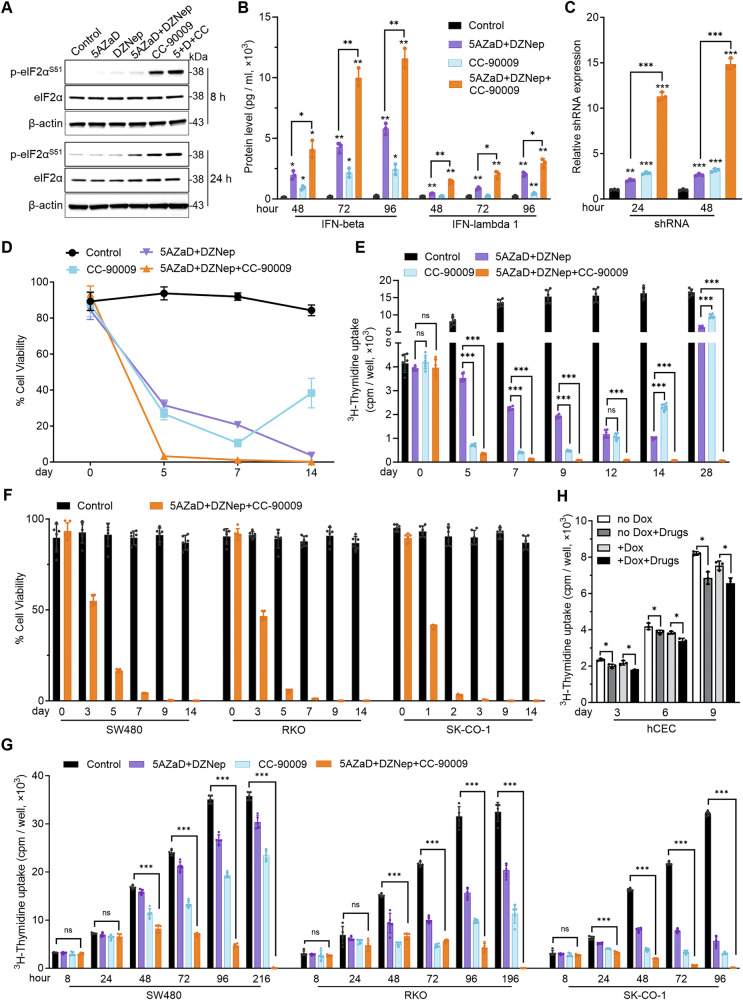
Effects of 5AZaD, DZNep and the ISR inducer CC-90009 on proliferation and viability of colon cancer cells. (**A**)Western blot analysis of ISR markers in R1+Dox treated with 5AZaD, DZNep, and CC-90009 individually or together (5 + D + CC: 5AZaD+DZNep+CC-90009) for 8 and 24 h (**h**). Control cells were incubated in culture medium with 0.1% DMSO. Representative experiment of 2 performed. (**B**) IFN-beta and IFN-lambda 1 protein levels (pg/ml) in culture supernatants of R1+Dox incubated in medium only (with 0.1% DMSO), with 5AZaD+DZNep, CC-90009, or 5AZaD+DZNep+CC-90009 for 48–96 h (h). Results from ELISA reflect the means (± SD; triplicate measurements). Representative of 4 experiments. (**C**)Relative shRNA expression in R1+Dox cells incubated in medium only (with 0.1% DMSO), with 5AZaD+DZNep, CC-90009, or 5AZaD+DZNep+CC-90009 for 24 or 48 h (h). Results from qPCR, normalized by RPL30, are displayed as relative to expression in medium (with 0.1% DMSO). The results reflect the means (± SD; triplicate measurements). Representative of 3 experiments. **D**, **E** Cell viability (**D**) and proliferation (**E**) of R1+Dox cultured in medium only (with 0.1% DMSO), with 5AZaD+DZNep, CC-90009, or 5AZaD+DZNep+CC-90009 for the indicated number of days. Viability (displayed as % viable cells/total) was measured by acridine orange/propidium iodide staining and proliferation by ^3^H-thymidine incorporation. The results reflect the means from triplicate measurements in D and 6 replicates in E ( ± SD). Results in (**D**) and (**E**) are representative of 3 experiments. **F** Cell viability of SW480, RKO, and SK-CO-1 colorectal cancer cells incubated in medium only (with 0.1% DMSO), or with 5AZaD+DZNep+CC-90009 for 0–14 days, measured by acridine orange/propidium iodide staining. The results, presented as % viable cells, reflect the means from six replicates (± SD). Representative of 3 experiments. **G** Proliferation of SW480, RKO, and SK-CO-1 colorectal cancer cells incubated in medium only (with 0.1% DMSO), with 5AZaD+DZNep, CC-90009, or with 5AZaD+DZNep+CC-90009 for the indicated number of hours; results from ^3^H-thymidine incorporation reflect the mean (± SD) cpm/six replicate cultures. Representative of 3 experiments. **H** Proliferation of the human immortalized colonic epithelial cell line CEC incubated with or without Dox, in medium only (with 0.1% DMSO) or with 5AZaD+DZNep+CC-90009; incubation time, 3-9 days; results from ^3^H-thymidine incorporation reflect the mean (± SD) cpm/triplicate cultures. Representative of 3 experiments. In this figure, 5AZaD (500 nM), DZNep (200 nM), CC-90009 (1 μM); error bars represent the mean ± SD from triplicate measurements, each point represents one replicate; *p* values from unpaired Student’s t-test are calculated relative to the untreated control; ns: not significant; *P < 0.05, **P < 0.01, ***P < 0.001.

Under experimental conditions showing limited ISR activation in R1+Dox by 5AZaD+DZNep, combining CC-90009+5AZaD+DZNep increased p-eIF2α^S51^ levels beyond those induced by either CC-90009 or 5AZaD+DZNep (Fig. 7A). Consistently, CC-90009+5AZaD+DZNep cooperatively promoted *ATF3*, *IFNB1* and *IFNL1* mRNA expression in R1+Dox, and to a lower degree in R1 no-Dox (Supplementary Fig. S7A–C). Also, CC-90009 + 5AZaD+DZNep significantly increased IFN-beta and IFN-lambda 1 secretion by R1+Dox (Fig. 7B), associated with increased chromatin accessibility at the *IFNB1* and *IFNL1* promoters (Supplementary Fig. S7D, E).

CC-90009+5AZaD+DZNep also cooperatively promoted expression of the integrated shRNA in R1+Dox (Fig. 7C), surpassing levels achieved in 596-7+Dox cells (Supplementary Fig. S5B). This effect of CC-90009+5AZaD+DZNep is reduced in R1 no Dox (Supplementary Fig. 8A). Notably, CC-90009 did not enhance chromatin accessibility to the shRNA mini-CMV promoter above that induced by 5AZaD+DZNep (Supplementary Fig. S8B), suggesting that CC-90009 acts as a posttranscriptional regulator of mRNA stability in this context [16, 30–33]. Also, functional experiments showed that CC-90009+5AZaD+DZNep produce a greater and more sustained reduction of cell viability and proliferation than achieved by CC-90009 or 5AZaD+DZNep (Fig. 7 D, E, and Supplementary Fig. S8C).

Next, we tested the effects of CC-90009+5AZaD+DZNep on the viability of human CRC cell lines with distinct genetic and epigenetic features [34]. In the SW480, RKO and SK-CO-1 cell lines, CC-90009+5AZaD+DZNep significantly reduced cell viability (Fig. 7F) and proliferation (Fig. 7G), while activating ISR, inducing expression of *ATF3*, *IFNB1* and *IFNL1* mRNAs, and increasing secretion of IFN-beta and IFN-lambda 1 (Supplementary Fig. S8D–F). Additionally, CC-90009 + 5AZaD+DZNep significantly reduced the proliferation in the HCT116, DLD1 and SW620 CRC lines, although to a lower degree than in the SW480, RKO and SK-CO-1 CRC cell lines (Supplementary Fig. S8G). Additionally, CC-90009+5AZaD+DZNep only modestly (albeit significantly (*p* < 0.05) reduced proliferation in the human telomerase immortalized colonic epithelial cell line, hCEC [35], which resembles “normal” colon-derived epithelial cells (Fig. 7H). Together, these culture results indicate that CC-90009+5AZaD+DZNep thwart growth and survival of R1 and other CRC lines.

### Anti-tumor activity of 5AZaD and DZNep combined with CC-90009

To evaluate the anti-tumor efficacy of combining epigenetic and ISR-inducing therapy, groups of NOD/SCID mice bearing R1 tumors and fed with Dox-containing chow were treated (i.p. injections, 3/week) with 5AZaD (1 mg/kg) plus DZNep (0.5 mg/kg) CC-90009 (2.5 mg/kg daily i.p.); 5AZaD (1 mg/kg) plus DZNep (0.5 mg/kg) plus CC-90009 (2.5 mg/kg); or formulation buffer only.

**Fig. 8:**
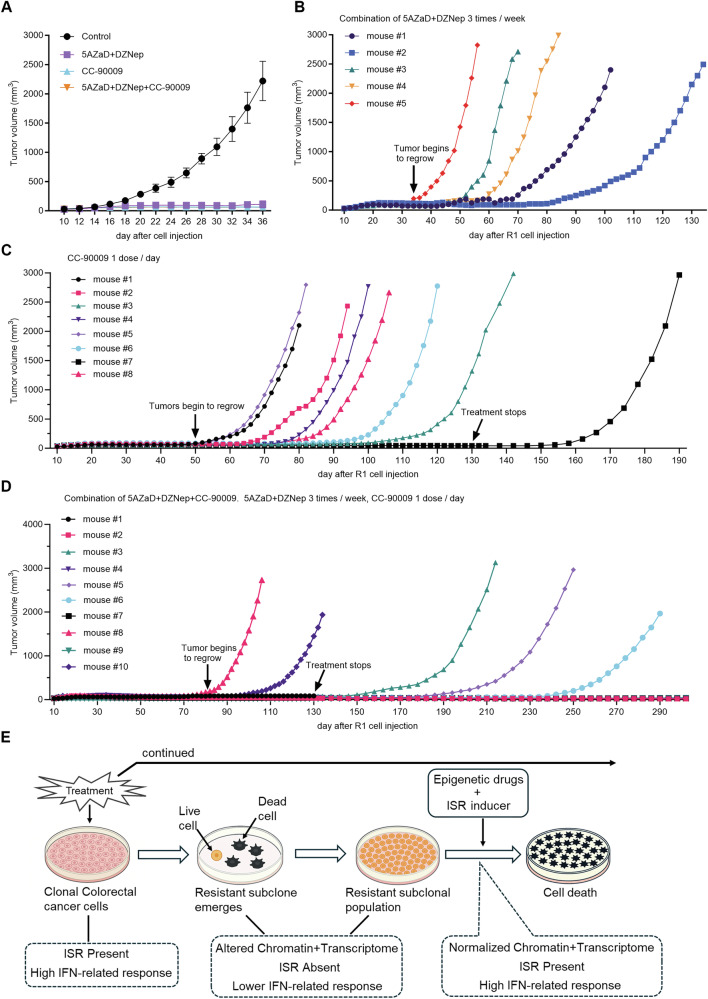
Combined epigenetic and ISR-targeting therapy can prevent outgrowth of R1 tumors. (**A**)Tumor growth of R1+Dox xenografts in NOD/SCID mice. R1 was injected s.c., and mice were fed doxycycline chow. On day 10, when tumors became measurable, mice were randomized to treatment with buffer only (Control *n* = 5), with 5AZaD (1 mg/kg)+DZNep (0.5 mg/kg; *n* = 5), with CC-90009 (2.5 mg/kg; *n* = 8) or the triple combination (*n* = 10) of 5AZaD (1 mg/kg)+DZNep (0.5 mg/kg)+CC-90009 (2.5 mg/kg) administered intraperitoneally three times per week for 5AZaD and DZNep, and daily for CC-90009. Tumor volumes represent mean ± SD at the indicated time points. **B–D** Individual tumor growth curves of R1+Dox xenografts in NOD/SCID mice treated with combination of 5AZaD+DZNep (**B**), or CC-90009 alone (**C**) or the triple combination of 5AZaD+DZNep+CC-90009 (**D**). The arrows show the time point that the tumor or tumors began to regrow. Tumors were measured starting on day 10 after inoculation. Each line represents a single animal. (**E**) Schematic illustration of the experimental results showing the emergence of a subclonal population of treatment-resistant cells from a clonal population of CRC cells experiencing ISR. The resistant population displays altered chromatin and transcriptome profiles. These alterations are normalized by epigenetic drugs plus an ISR-inducing drug, which together prompt CRC cell death. IFN levels are high when ISR is present but are detected at lower levels in the resistant CRC cell population.

All drug-regimens controlled tumor growth over a 36-day period, whereas all control mice reached the tumor size-endpoint by day 36 (Fig. 8A). We continued treatment in all treatment groups. All mice in the 5AZaD+DZNep group developed tumors within 80 days and reached tumor size-endpoint by day 134 (Fig. 8B). Most mice (7/8) in the CC-90009 treatment group developed a tumor at a variable time-point between day 50 and day 106; one mouse remained tumor free until day 150, with no treatment since day 130 (Fig. 8C).

In the 5AZaD+DZNep+CC-90009 treatment group, only two mice (2/10) developed a tumor by day 130 (Fig. 8D). One mouse, without tumor, developed a respiratory syndrome prompting humane euthanasia on day 130. Although all other mice appeared healthy based on body weight (Supplementary Fig. S8H), appearance, and activity, we stopped drug treatment on day 130 in all mice (*n* = 7). Observation off drugs detected tumor emergence in three additional mice on days 154, 190 and 246 (Fig. 8D). The four remaining mice are tumor-free as of this writing, day 302, having received no treatment for 172 days. These results indicate that the drug combination of 5AZaD+DZNep+CC-90009 is effective at significantly delaying colon carcinoma tumor growth and even eradicating colon carcinoma tumors in mice.

Overall, these results show (Fig. 8E) that acute stress induces ISR in a clonal population of CRC cells, associated with high-level IFN production, death of most cells, and emergence of a subclonal resistant cell population with altered chromatin and transcriptome landscape, and low-level IFN production. Epigenetic drugs combined with an ISR inducer cause ISR associated with high-level IFN production, normalize chromatin and transcriptome, and promote massive cell death.

## Discussion

Since CRC progression frequently arises in the absence of new genetic alterations, epigenetic mechanisms are proposed to play a critical role [10]. Here, modeling clonal CRC, we have characterized subclonal diversity as a key driver of therapeutic resistance and linked this diversity to a broad reprogramming of the epigenome and transcriptome. This epigenomic and transcriptional reprogramming was linked to the presence of a low-level and persistent interferon response, which arose after the clonal CRC cell population was stressed, albeit a causal relationship between IFN signaling and altered chromatin landscape in the resistant cells is still unclear. We find that inhibitors of DNA and histone methylation combined with a GSPT1-degrader can correct the epigenetic and transcriptional reprogramming of the resistant population and block CRC progression in mice. In sum, our study describes a new mechanistic approach to reverse a resistant CRC phenotype.

The current study relied on shRNA-induced stress. However, metabolic stress [36], oxidative stress [37], endoplasmic reticulum stress [38], and chemotherapy induced genotoxic stress [39, 40] are common in cancer cells. Activation of PKR [41, 42], ISR [43], and IFN [44, 45] signaling are also common to diverse cancers, indicating that the model explored in this study reflects tumor cell states arising from diverse stress signals imposed on cancer cells.

An early phase II clinical trial in heavily-pretreated CRC patients combining 5AZaD with the histone deacetylase inhibitor, entinostat, detected reversal of DNA hypermethylation in a subset of patients with improved progression-free survival, but no significant overall clinical activity [46]. To improve on epigenetic treatment, we successfully exploited CC-90009, a cereblon-dependent GSPT1degrader that induces ISR and transcriptome reprogramming [15, 16, 47–49]. The drug was well tolerated in NOD/SCID mice, with the important caveat that mice carry a mutant cereblon [50], such that the xenografted human-derived R1 tumors are the only drug targets in this system. CC-90009 has undergone initial clinical testing in acute myelogenous leukemia, but the trial was terminated due to “lack of efficacy in the short-term acute phase” (https://clinicaltrials.gov/study/NCT02848001), but other ISR activators have shown potential as cancer therapeutics in preclinical cancer models [51–53].

The ISR has been linked to promoting tumorigenesis and tumor progression, in part through T-cell immune evasion [54, 55]. Our results, showing that ISR initially promoted cancer cell resistance and later reversed the resistant phenotype highlights a key property of ISR to induce either cell survival or cell death. IFNs display a similar dual role in cancer [44]. Strong and acute IFN response are cytotoxic, whereas weak and chronic responses promote cell survival and confer treatment resistance [45]. We speculate that improved understanding of ISR- and IFN-induced pathways could inform how to rationally exploit ISR and IFN for anti-cancer treatment.

Some studies emphasized the tumor microenvironment as a determinant of cancer plasticity and drug responses [56–60]. Our results show that therapy can directly induce transcriptional reprogramming and subclonal diversity in CRC. Thus, tumor cell shifts from one state to another can be cell autonomous. Other studies identified the histone methyltransferase enzyme MLL3 [61] and the chromatin-remodeling enzyme, ATRX [14] as crucial regulators of cancer plasticity. The current study points to the contribution of multiple epigenetic modifiers to subclonal transcriptome plasticity associated with ISR [15, 16, 47–49]. Collectively, our study sheds light on the mechanisms that drive cellular plasticity in CRC, how these processes contribute to cancer progression and provide an opportunity for effective therapeutic intervention.

## Materials And Methods

### Cells, cell lines, assays for cell death, viability, protein content, and proliferation

We used the human colorectal cancer (CRC) cell lines from the American Type Culture Collection (ATCC, Manassas, VA, USA) HT29 (Cat# HTB-38), HCT116 (Cat# CCL-247), DLD1 (Cat# CCL-221), SW480 (Cat# CCL-228), SW620 (Cat# CCL-227), RKO (Cat# CRL-2577), SK-CO-1 (Cat# HTB-39), and the human kidney HEK 293 T cell line (Cat# CRL-3216). We also used an immortalized human colon epithelial cell line (hCEC) [35] provided by the originator, Dr. Jerry W. Shay, and the clonal CRC cell line designated 596-7, which carries a doxycycline (Dox)-inducible EFNB2-targeting shRNA expression cassette in a TRIPZ lentiviral vector (Thermo Fisher Scientific Open Biosystems, Huntsville, AL, USA) [19]. A sub-clonal cell line derived from 596-7, identified as R1, was generated during this study as detailed in the manuscript and Supplementary Materials and Methods.

All cells, mycoplasma negative, were cultured under standard conditions (37 °C, 5% CO₂) in the appropriate growth media with supplements, as detailed in Supplementary Materials and Methods. Assays to evaluate cell death, cell viability, cell proliferation, and cellular protein content were performed as described [62, 63] and detailed in the Supplementary Materials and Methods. In brief, cell death was quantified by Annexin V/DAPI staining and flow cytometry, cell viability was assessed by acridine orange/propidium iodide live-dead staining, cell proliferation was measured by ³H-thymidine incorporation, and total protein content was determined by a colorimetric assay. Primers and primer sequences are shown in Supplementary Table S1; and information on all antibodies (sources and dilutions) is available in Supplementary Table S2.

### DNA extraction and sequencing, RNA extraction, qPCR, ChIP-qPCR, RNA-seq, Bisulfite sequencing, and FAIRE-qPCR

Standard protocols were used for nucleic acid and chromatin analyses. Detailed procedures for total RNA extraction, cDNA synthesis and quantitative real-time PCR (qPCR), chromatin immunoprecipitation followed by qPCR (ChIP–qPCR), bisulfite DNA conversion and sequencing, and high-throughput RNA sequencing (RNA-seq) are provided in Supplementary Materials and Methods. Formaldehyde-assisted isolation of regulatory elements (FAIRE) was performed to assess chromatin accessibility, essentially as described [20, 64]. Briefly, cells were crosslinked by treatment with 1% formaldehyde, then glycine was added to quench the crosslinking. Nuclei were isolated, and chromatin was sheared by sonication using a Misonix Sonicator 3000 (Misonix, Farmingdale, NY, USA) to obtain DNA fragments of ~200–500 bp. The sheared chromatin was subjected to phenol–chloroform extraction (Sigma-Aldrich, St. Louis, MO, USA; Cat# 77617). DNA in the aqueous phase (enriched for “free DNA” from nucleosome-depleted regions) was recovered by ethanol precipitation and resuspended in TE buffer (Thermo Fisher Scientific, Waltham, MA, USA; Cat# 12090015). The abundance of accessible chromatin at selected loci was then quantified by qPCR, and results were expressed as the ratio of free DNA to total input DNA. See Supplementary Materials and Methods for FAIRE primer sequences and additional details.

### CRISPR/dCas9 shRNA activation

To activate the inducible shRNA in R1 cells, which are poorly sensitive to Dox activation, we employed a CRISPR-based system using a nuclease-deficient Cas9 fused to a VP64 transcriptional activator (dCas9-VP64). Cells were first transduced with a lentiviral vector encoding dCas9-VP64 (Addgene plasmid #61425; Addgene, Watertown, MA, USA). After 24 h, the cells were transduced with a second lentivirus carrying single-guide RNAs (sgRNAs) targeting the minimal CMV promoter upstream of the shRNA cassette (three sgRNAs: #829, #830, #831) or a non-targeting control sgRNA (#883). Details of the sgRNA vector design and transduction protocol are provided in the Supplementary Materials and Methods.

Four days after sgRNA transduction, induction of the EFNB2 shRNA was evaluated by the expression of the red fluorescent protein (RFP) reporter (visualized by fluorescence microscopy using an Olympus IX51 inverted microscope; Olympus Corp., Tokyo, Japan) and by pPCR quantification of the shRNA transcript.

### Mouse xenograft studies

All animal experiments were approved by the Institutional Animal Care and Use Committee of the Center for Cancer Research (CCR, Bethesda, MD), NCI, NIH, and were conducted according to the NIH Guide for the Care and Use of Laboratory Animals (National Academies Press, 2011). Female NOD/SCID mice (6–8 weeks old; Jackson Laboratory; Bar Harbor, ME, USA; 001303) were individually injected s.c. with 10 × 10⁶ cancer cells and fed Dox-containing chow (Bio-Serv, Flemington, NJ; S3888, 200 mg/kg). When tumors became measurable, mice were randomized to receive 5AZaD (1 mg/kg i.p., three times/week.), DZNep (0.5 mg/kg i.p., three times/week), CC-90009 (2.5 mg/kg i.p., once/day), or drug combinations (at the same concentrations used individually and with the same frequency). Mouse group assignment was based on tumor size such that the average tumor size/group was not significantly different from control by unpaired Student’s t test. Sample size was estimated based on our published experiments and preliminary drug-dose finding experiments. No mouse was excluded from analysis. The investigator was not blinded to the group allocation during the experiment. All drugs were from DPT Chemical Repository, NCI, Rockville, MD). Tumor Volume (V) was calculated from caliper measurements as π/6 x L x W x W; L: longest diameter, W: perpendicular to L, π = 3.1416.

### Statistical analyses

GraphPad Prism (GraphPad Software, version 10, San Diego, CA, USA) was used to perform statistical tests. Results are presented as means ± standard deviations with experimental and technical replicates indicated. Fisher’s exact test was used to calculate the statistical significance of enriched IPA and GO pathways; unpaired two-tailed Student’s t test was used for analysis of two groups with homoscedastic distribution; *p-*values < 0.05 were considered statistically significant. The parametric statistical tests were applied to normally distributed data, with acceptable estimates of variance within each group and between groups.

## Supplementary information

Supplemental Material

Supplemental Tables 1 and 2 in editable format

## Data Availability

The RNA-seq data for this study can be accessed at GEO repository at NCBI https://www.ncbi.nlm.nih.gov/geo/query/acc.cgi?&acc=GSE312823 under GEO accession Number: GSE 312823. The whole genome sequencing (WGS) fastq files generated in this study have been submitted to the SRA of NCBI *(*https://www.ncbi.nlm.nih.gov/bioproject/*)* under accession number PRJNA1377467. All codes used in this paper are available at https://github.com/kopardev/ccbr1060. The approved mouse protocol supporting the mouse experiments, the reagents and data generated in this study are available upon request to the corresponding author.
