# Supplementary material for: Targeting the epigenome and the integrated stress response to normalize colorectal cancer subclonal plasticity and progression
Source: Cell Death Dis. 2026 Apr 10;17(1):459. doi: 10.1038/s41419-026-08720-2 (PMC13181133; doi:10.1038/s41419-026-08720-2)
Supplement: Supplementary file 1 — Supplemental Material [file 41419_2026_8720_MOESM1_ESM.pdf]

**Supplementary Data for**

# Targeting the epigenome and the integrated stress response to normalize colorectal cancer subclonal plasticity and progression

Lili Li, Taekyu Ha, Jing-Xin Feng, Michael DiPrima, Dunrui Wang, Parthav Jailwala, Thomas Meyer, Justin Lack, Hidetaka Ohnuki, and Giovanna Tosato

**Contents:**

1. Supplementary Materials and Methods with References
2. Supplementary Tables
3. Supplementary Figures and Legends

# Supplementary Materials and Methods

## Cells, cell culture, and reagents

The HT29 (ATCC, HTB-38) and HCT116 (ATCC, CCL-247) cell lines were grown in McCoy5A medium (Gibco, 16600082) supplemented with 10% FBS (Sigma-Aldrich, F2442) and penicillin/streptomycin (Gibco, 15140-122); the DLD1 (ATCC, CCL-221) cell line was grown in RPMI-1640 medium (Corning, 10-040-CV), supplemented with 10% FBS and penicillin/streptomycin; the SW620 (ATCC, CCL-227), SW480 (ATCC, CCL-228), RKO (ATCC; CCL-2577),  SK-CO-1 (ATCC, HTB-39) and 293T (ATCC, CRL-3216) cell lines were grown in DMEM (Gibco, #11965092), supplemented with 10% FBS and penicillin/streptomycin (1). The immortalized colon epithelial cells hCEC (a gift from Dr. Jerry W Shay)(2) was grown in DMEM, supplemented with EGF (25 ng/mL; Gibco, AF-100-15), hydrocortisone (1 µg/mL; Sigma, H0888), insulin (10 µg/mL)-transferrin (2 µg/mL)-sodium selenite (5 nM) (Sigma, I1884), 2% newborn calf serum (Sigma, N4637), and gentamicin sulfate (50 µg/mL; Sigma, G1914). The clonal 596-7 cell line was maintained in McCoy's 5A medium containing 10% tetracycline-negative FBS (Corning, 35-075-CV), 1% penicillin/streptomycin, and 3 µg/mL puromycin (Gibco; A11138-03). All cells were cultured at 37°C in a humidified incubator with 5% $CO_2$.

In cell culture, Doxycycline (Dox; Sigma, D9891) was used at 1 µg/mL to induce or maintain shRNA expression; 5-Aza-2′-Deoxycytidine (5AZaD; Sigma, 189826) was used at 500 nM, 3-deazaneplanocin A (DZNep; Sigma, 252790) was used at 200 nM, and the ISR inducer CC-90009 (a gift of Dr. Steven White) was used at 1 µM. All these drugs were reconstituted in

DMSO; the final DMSO concentration in the cultures was 0.1%. Diluent control was adjusted to the DMSO concentration of 0.1%.

**Generation of the inducible and resistant cell lines**

The generation and characterization of the 596-7 cells expressing an *EFNB2*-targeting shRNA using the TRIPZ vector (Thermo Scientific Open Biosystems; RHS4750) with a mini-CMV promoter and RFP reporter was previously reported by Ha et al. (3). Expression of the shRNA in 596-7 cells (induced by 1 μg/mL Doxycycline) caused rapid death of most cells. The surviving cells grew to generate a derivative cell line by day 7 designated as the R1 cell line that was maintained in the McCoy's 5A medium containing 10% tetracycline-negative FBS, 1% penicillin/streptomycin, 3 μg/mL puromycin and 1 μg/mL Doxycycline.

**DNA sequencing**

To validate the integrity of the *EFNB2*-targeting shRNA, genomic DNA from R1 cells was Sanger-sequenced using the primer (5′-CAAACTGGGGCACAGATAATC-3′), which anneals within the shRNA cassette. The resulting Sanger read spanned the entire integrated EFNB2 shRNA cassette, and its sequence was aligned to the original pTRIPZ-CMV-EFNB2-shRNA plasmid, revealing a perfect match to the intended shRNA insert.

**Generation of pTRIPZ-EFNB2-Tet-on shRNA and Lentivirus production**

The procedures for shRNA cloning were as described (3). The human *EFNB2* shRNA$^{miR}$ cassette, designed as a miR-30-based shRNA vector, was cloned into the pTRIPZ vector. Identity of the integrated shRNA was confirmed by Sanger sequencing.

Lentiviral particles were produced as described (4). Briefly, 293T cells were co-transfected with a third-generation packaging system, the lentiviral transfer vector together with the packaging plasmids psPAX2, and vesicular stomatitis virus G protein expression plasmid pMD2.G, using a standard Lipofectamine 2000-based transfection protocol (Thermo Fisher Scientific, 11668019). Virus-containing supernatants were collected at 48- and 72-hours post-transfection from the culture medium, cleared by low-speed centrifugation and filtration (0.45 µm), and concentrated by ultracentrifugation (SW28 rotor, 19,500 rpm, 140 min at 4 ºC), and stored at -80ºC until use. The virion-containing pellet was resuspended in an appropriate volume of complete medium (DMEM supplemented with 10% FBS). Viral titer, expressed as Infectious Units (IU), was quantified by qPCR Lentivirus Titer Kit (Biological Materials, LV-900).

**Cell death**

Cell death was assessed by flow cytometry using a BD LSRFortessa instrument (BD Biosciences, San Jose, CA, USA). Cells were harvested, washed twice with cold PBS, and suspended in 1× Binding Buffer (BioLegend, 422201) at a concentration of $1 \times 10^6$ cells/ml. After transfer 100 µL of cell suspension in 5 mL test tube, cells were stained with 5 µL APC/Fire 750-Annexin V (BioLegend, 640953) and 30 µM DAPI (BioLegend, 422801). The results were analyzed with FlowJo v10 software (BD Biosciences, Ashland, OR, USA).

**Cell viability assay**

R1 cells were seeded at $4 \times 10^3$ cells per 6-cm dish in 6 mL complete medium and allowed to attach overnight. Drug treatments were initiated the following day. To maintain drug activity and prevent nutrient depletion during long-term culture, one-half of the culture medium

(3 mL) was removed every 48 hours and replaced with 3 mL of fresh medium containing 2× drug concentrations, resulting in the correct final drug concentration. All treatment groups underwent identical medium-replacement steps.

At each indicated time point, cell viability was assessed by adding acridine orange/propidium iodide (AO/PI) double staining (Fisher Scientific, NC1765655) directly to the culture dish to achieve a final concentration of 100 μg/mL acridine orange and 100 μg/mL propidium iodide. Cultures were incubated for 5 minutes at room temperature in the dark before imaging. This approach preserves both adherent and floating dead cells and thus reflects the proportion of viable versus non-viable cells at each time point, rather than cumulative cell death over the entire period. Stained cells were visualized using a fluorescent microscope (Olympus; IX51, Tokyo, Japan). Live cells exhibited green fluorescence (AO-permeable), dead or membrane-compromised cells showed red fluorescence from PI uptake. At least five randomly selected fields per sample were imaged, and viable versus non-viable cells were quantified using ImageJ 1.54 software with threshold-based fluorescence separation. Results are expressed as the percentage of viable (AO-positive, PI-negative) cells relative to the total cell population.

**Cell proliferation assays**

Cell proliferation was assessed by $^{3}$H-thymidine incorporation (PerkinElmer) as described (3). Briefly, $^{3}$H-thymidine (0.5 μCi, Perkin Elmer, #NET027WW0011MC) was added to cell cultures (200 μL, 96-well flat-bottom plates) for 8 hours, and then the culture plates were frozen (-80 °C). After defrosting, pulsed cells were collected onto glass fiber filters (Perkin Elmer, #1450-421), and incorporated radioactivity was counted in a liquid scintillation counter (Perkin Elmer; MicroBeta-1450, Waltham, MA, USA).

**CRISPR/dCas9 shRNA activation**

To generate sgRNA-expressing lentiviral vectors, sgRNAs oligonucleotides targeting the mini-CMV promoter (sgRNA #829, #830, #831) or a non-targeting control (sgRNA #883) were annealed and inserted into the pXPR_502-based lentiviral vector (Addgene #96923). Lentiviral particles carrying sgRNA-expressing constructs were produced as described (4).

To induce shRNA activation, cells were seeded ($1{\times}10^5$ cells/well) in 12-well plates (Corning, 353043) and transduced with 10 µL ($1.5 \times 10^8$ IU/µL) of lentivirus-containing solution expressing dCas9-VP64 (Addgene #61425); 24 hour later, dCas9-VP64–expressing cells were infected with 10 µL ($1.7 \times 10^8$ IU/µL) of sgRNA lentivirus-containing solution in complete medium. After infection, cells were incubated for four days to allow expression of sgRNAs and activation of the shRNA cassette in the pTRIPZ system. RFP expression was evaluated by fluorescent microscopy, and shRNA induction was quantified by qPCR using primers specific to the mature shRNA transcript. Differential interference contrast (DIC) and red fluorescence images were acquired with an Olympus inverted microscope (Olympus; IX51).

**Western blotting**

Cells were lysed in lysing buffer [50 mM Tris (pH 7.4), 150 mM NaCl, 1% Triton X-100, 1 mM EDTA, 1 mM EGTA, 1 mM α-glycerophosphate, 2.5 mM sodium pyrophosphate, 1 mM $Na_3VO^4$, 10 mM NaF, and 1x protease inhibitor (Roche, # 11836153001)]. Cell lysates were separated by sodium dodecyl sulfate polyacrylamide gel electrophoresis (SDS-PAGE), transferred to Amersham Protran membranes (Cytiva, #10600001), membranes were blocked with blocking buffer (Thermo Fisher Scientific, 37515), incubated with primary and secondary

antibodies, and visualized by Pierce™ ECL Western Blotting Substrate (Thermo Fisher Scientific, 32109). Antibodies used are listed in Supplementary Table S2.

**ELISA**

Secreted IFN-beta and IFN-lambda 1 were quantified in cell culture supernatants with the Quantikine ELISA kit (R&D Systems, DIFNB0) and the DuoSet ELISA kits (R&D Systems, DY7246), respectively, according to the manufacturer's instructions. The colorimetric reaction was measured at 450 nm using a microplate reader (Imgen Technologies, BMG LABTECH, Ortenberg, Germany). Samples were diluted 1:20 in base culture medium before ELISA, and final concentrations were corrected for the dilution factor.

**Formaldehyde-Assisted Isolation of Regulatory Elements (FAIRE)**

FAIRE was performed to isolate nucleosome-depleted, transcriptionally accessible chromatin regions as previously described (5). Briefly, 596-7 or R1 cells cultured under the conditions described, were crosslinked by adding formaldehyde (final concentration 1%) directly to the culture medium for 7 minutes at room temperature. Crosslinking was quenched by the addition of 125 mM glycine for 5 minutes, followed by twice washing with cold PBS.

Cell pellets were resuspended in FAIRE-lysis buffer (1% SDS, 10 mM EDTA, 50 mM Tris-HCl, pH 8.1) and incubated on ice for 10 minutes. Chromatin was sheared by Misonix Sonicator 3000 (Misonix Inc., Farmingdale, NY, USA) for a total time of 5 min, alternating 20-second in ice water followed by 10-second sonication (level 1.5), to yield DNA fragments in the 200–500 bp range. After centrifugation (15,000g for 5 min at 4 °C), 10% input from supernatant was reverse crosslinked with 20 µg RNaseA (Thermo Fisher Scientific, 12091) for 30 min at

37 °C, and 1 µL proteinase K (Qiagen, 19134) for 60 min at 55 °C, and then overnight at 65 °C. The remaining cell lysate (FAIRE DNA) and de-crosslinked input DNA were subjected to phenol–chloroform extraction using phenol-chloroform-isoamyl alcohol (25:24:1, Sigma, P3803). FAIRE DNA was then reverse crosslinked as described above.

DNA was then purified by ethanol precipitation. DNA pellets were washed with 70% ethanol, air-dried, and resuspended in TE buffer. Total input DNA from sonicated cells was processed in parallel prior to organic extraction to allow for normalization. The abundance of accessible chromatin was assessed by qPCR using primers targeting mini-CMV, *FAS*, *ATF3*, *IFNB1*, *IFNL1*, *ACTB* and *RPL30* gene promoters (see Supplementary Table S1). FAIRE enrichment was quantified as the ratio of FAIRE DNA (free DNA) to input DNA (total DNA).

**RNA extraction and qPCR**

Total RNA was extracted using the RNeasy kit (Qiagen; 74106); 1 mg RNA was used to synthesize cDNA with the QuantiTect reverse transcription kit (Qiagen; 205311). RNA integrity (RIN) was evaluated using the Bioanalyzer 2100 (Agilent Technologies, Santa Clara, CA, USA). mRNA expression was measured (7900HT Fast Real-Time PCR system, Biosystems, Foster City, CA, USA) using the FastStart Universal SYBR Green Master Mix (ROX) (Roche, 04913914001). Relative mRNA expression was calculated using the ΔΔCt method (ΔCt=Ct[gene of interest] - Ct[RPL30]; ΔΔCt = ΔCt[treated sample] – ΔCt [untreated sample]). Ct (cycle threshold) values were calculated by SDS 2.4.1 software (Applied Biosystems). Expression values were normalized to RPL30. The list of primers is provided in Supplementary Table S1.

**ChIP-qPCR**

Chromatin immunoprecipitation (ChIP) was performed using the SimpleChIP® Enzymatic Chromatin IP Kit, following the manufacturer's protocol (Cell Signaling Technology, #9003). Briefly, R1 cells were crosslinked with 1% formaldehyde for 10 min at RT, and the cross-linking was quenched with 125 mM glycine for 5 min at RT. After nuclei extraction, chromatin was sheared by Misonix Sonicator 3000 (Misonix Inc., Farmingdale, NY, USA) for a total time of 3 min; alternating 30-second in ice water followed by 20-second sonication, at level 1.5 to yield chromatin-DNA into 200–500 bp fragments suitable for immunoprecipitation. These chromatin fragments were incubated with rabbit antibodies to H3K4me3, H3K9me3 and H3K27me3 (listed in Supplementary Table S2), or with rabbit IgG control (included in the kit) to assess background binding. Antibody-bound chromatin complexes were isolated by precipitation using Protein A/G Magnetic beads, washed, and eluted. Crosslinks were reversed by using RNase A and Proteinase K. The purified DNA was analyzed by qPCR using *FAS* promoter primers (Supplementary Table S1). qPCR signals were first normalized to input DNA, and then fold enrichment of each target was calculated relative to the IgG control and to total histone H3 levels (also normalized by input DNA and control IgG).

**Bisulfite sequencing**

596-7 and R1 cells were washed with PBS, scraped, and pelleted by centrifugation (1000 rpm, 2 min). Cell pellets were resuspended in PBS and genomic DNA was isolated and purified using the DNeasy Blood and Tissue Kit (Qiagen, Cat# 69504) following the manufacturer's instructions. Genomic DNA (500 ng) was subjected to bisulfite conversion using the EpiJET Bisulfite Conversion Kit (Thermo Fisher Scientific, Cat# K1461) following the manufacturer's

instructions. Converted DNA was eluted in 25 µL of nuclease-free water and stored at −20°C. Bisulfite-converted DNA was PCR amplified using a primer pair specific for the CpG-rich regions upstream of the *FAS* promoter (BSP-fw: 5′-GAGAGGTTGTGAGGTGGGTGTA-3′; BSP-rev: 5′-TAACATAAAATAACTTCACAAAC-3′) with the bisulfite-compatible hot-start polymerase HotStarTaq DNA Polymerase (Qiagen, Cat# 203203). PCR conditions: 95°C 15 min; 40 cycles (94°C for 30 sec, 55–60°C for 30 sec, and 72°C 45 sec) followed by 72°C for 10 min and then 4°C. PCR products were verified by agarose gel electrophoresis. PCR amplicons were gel-purified using QIAquick Gel Extraction Kit (Qiagen, 28706) and cloned into the pCR™2.1-TOPO™ vector using the TOPO™ TA Cloning Kit with One Shot™ TOP10 competent *E. coli* and PureLink™ Quick Plasmid Miniprep Kit (Thermo Fisher Scientific, Cat# K450002), following the manufacturer's protocols. After transformation, individual colonies were picked and grown overnight. Plasmid DNA was isolated using the PureLink™ Quick Miniprep Kit, and inserts were sequenced using the M13 Forward and M13 Reverse primers supplied with the kit. For each clone (8-11 independent clones obtained), CpG sites were scored as "methylated" (displayed as filled circles), cytosine retained after bisulfite conversion, or "unmethylated" (displayed as open circles), cytosine converted to thymine. Methylation levels at each CpG site were calculated as the proportion of methylated clones/sequenced clones. Methylation frequency at each CpG site was calculated as:

$$\text{Methylation \%} = \frac{\text{number of methylated clones}}{\text{total clones sequenced}} \times 100$$

**RNA sequencing and analysis**

RNA samples for RNA-seq had an RNA Integrity Score higher than 9.9. A stranded total RNA library was prepared according to the Illumina library prep protocol (Illumina; document

1000000040499 v.00). Pair-end stranded RNA-seq was performed by the Sequencing Facility at the Frederick National Laboratory for Cancer Research using Illumina Nextseq (596-7 no Dox, 596-7+Dox and R1+Dox). RNA-seq was performed in 596-7 cells cultured without (596-7 no Dox) or with Dox for 24 hours (596-7+Dox) and in R1 cells cultured with Dox for 4 months. The results of RNA-seq are deposited at https://www.ncbi.nlm.nih.gov/geo/. Normalized counts per million using trimmed mean of M-values (CPM-TMM) were used for analyses. Fold changes were calculated for R1 + Dox vs. 596 no Dox (Figure 2A, 2C, 2D and Supplementary Figure 2A and 2B), R1 + Dox vs. 596 + Dox (Figure 2B).

Differentially expressed genes from the RNA-seq datasets were used for downstream pathway enrichment analyses with Ingenuity Pathway Analysis (IPA; QIAGEN, https://digitalinsights.qiagen.com/) and Gene Ontology (GO). For both analyses, the input gene set consisted of significantly regulated genes fold-change > 1.3 and p < 0.05) between the two experimental groups.

For IPA, the Core Analysis workflow was performed using the Ingenuity Knowledge Base as the reference set. Canonical pathways, upstream regulators, and downstream biological functions were evaluated using Fisher's exact test, with significance determined after multiple-testing correction. The enrichment shown as % DEGs / pathway and –log(*p*-value) were defined from the IPA software's standard pathway statistics.

For GO analysis, differentially expressed genes were submitted to the PANTHER Overrepresentation Test using the GO Biological Process category and the *Homo sapiens* reference gene list. Statistical significance was assessed using Fisher's exact test with false discovery rate (FDR) correction. The *p*-values were taken directly from the PANTHER output and used for visualization. We additionally calculated a "% DEGs / pathway" for each GO term,

defined as the number of DEGs assigned to that term divided by the total number of genes included in that GO term.

**Whole-genome sequencing and variant analysis**

Genomic DNA from R1 and 596-7 cells was prepared using the TruSeq Sample Prep Kit (Illumina, IP-202-1012) and sequenced on an Illumina HiSeq 4000 instrument (Illumina Inc., San Diego, CA, USA) to generate 150-bp paired-end reads (2 × 150 cycles). Base calling was performed using Illumina RTA v2.4.6, and demultiplexing was done with bcl2fastq v2.17. Adapter trimming and quality filtering were performed using Trimmomatic v0.36. Trimmed reads were aligned to the human reference genome (hg38) using Bowtie2 v2.2.6. BAM file manipulation and duplicate marking were performed with Picard MarkDuplicates. Variant calling was performed using a somatic mutation-calling pipeline based on MuTech2 (Broad Institute Gene Analysis Toolkit). Only variants with sequencing depth ≥10× and passing the pipeline's internal quality filters were retained. Variants were annotated using the Ensembl Variant Effect Predictor (VEP) and cross-referenced with the COSMIC database. Variant calling was performed for each biological replicate of R1 and 596-7 cell lines, and the resulting variant lists were used to summarize mutation categories, including known COSMIC mutations, coding-sequence (CDS) mutations, splice-region mutations, and newly detected mutations. Unique mutations in R1 or 596-7 cells were identified by comparing the annotated variant lists between the two cell lines. Mutation frequency for each variant was calculated as the ratio of mutant read counts to the total sequencing depth at each genomic position.

**In vivo xenograft studies**

For generation of tumors, 10×10$^6$ 596-7 or 10×10$^6$ R1 cells were resuspended in 100 µL PBS and injected subcutaneously into the flanks of female NOD/SCID mice (6–8 weeks old; Jackson Laboratory, 001303). All mice were fed doxycycline-containing chow. Once tumors were measurable, mice were randomized into treatment groups.

In the vivo experiment shown in Figure 6C and D, 5AZaD was administered at 1 mg/kg intraperitoneally (i.p.) three times per week; DZNep was administered at 0.5 mg/kg i.p., three times per week; both compounds were dissolved in sterile saline (0.9% NaCl).

In the vivo experiment shown in Figure 8A-D, 5AZaD was administered at 1 mg/kg i.p. three times per week; DZNep was administered at 0.5 mg/kg i.p., three times per week; CC-90009 was administered at 2.5 mg/kg i.p., once daily; all compounds were dissolved in a formulation buffer composed of 50% sterile saline, 45% PEG400 (Sigma, 8074850050), and 5% NMP (Sigma, 443778), as described (6). When administered together, the drugs were mixed into a single 100 µL injection volume. Control animals received vehicle injections, either saline (Figure 6C and D) or formulation buffer (Figure 8A-D).

Tumor growth and body weight were monitored every two days in the experiment shown in Figure 6C and D; in the experiment shown in Figure 8A-D, tumor growth and body weight were monitored every two days before day 134 and every four days after day 134. Mice were euthanized when tumors reached or approached a size of 20 mm in any direction or developed a humane endpoint for euthanasia, following approved animal protocols.

**References**

1. DiPrima M, Wang D, Troster A, Maric D, Terrades-Garcia N, Ha T*, et al.* Identification of Eph receptor signaling as a regulator of autophagy and a therapeutic target in colorectal carcinoma. Mol Oncol **2019**;13:2441-59

2. Roig AI, Eskiocak U, Hight SK, Kim SB, Delgado O, Souza RF*, et al.* Immortalized epithelial cells derived from human colon biopsies express stem cell markers and differentiate in vitro. Gastroenterology **2010**;138:1012-21 e1-5
3. Ha T, DiPrima M, Koparde V, Jailwala P, Ohnuki H, Feng JX*, et al.* Antisense transcription from lentiviral gene targeting linked to an integrated stress response in colorectal cancer cells. Mol Ther Nucleic Acids **2022**;28:877-91
4. Salvucci O, Ohnuki H, Maric D, Hou X, Li X, Yoon SO*, et al.* EphrinB2 controls vessel pruning through STAT1-JNK3 signalling. Nat Commun **2015**;6:6576
5. Simon JM, Giresi PG, Davis IJ, Lieb JD. Using formaldehyde-assisted isolation of regulatory elements (FAIRE) to isolate active regulatory DNA. Nat Protoc **2012**;7:256-67
6. Surka C, Jin L, Mbong N, Lu CC, Jang IS, Rychak E*, et al.* CC-90009, a novel cereblon E3 ligase modulator, targets acute myeloid leukemia blasts and leukemia stem cells. Blood **2021**;137:661-77

**Supplementary Table S1. Primer sequences for qPCR, FAIRE-qPCR, and ChIP-qPCR**

| Application | Target gene | Forward primer (5′–3′) | Reverse primer (5′–3′) |
|---|---|---|---|
| qPCR | EFNB2 | AGCTGCAGATGGTCATTTCC | CTGGTGTGGTTCTGGTTCTG |
| qPCR | ATF3 | CTTGCCACTTTTTCCTTGGT | TGCTTGTGGGGAAGTTTGAT |
| qPCR | IFNB1 | CAACTGCAAGCCAATTGTTC | TGCTGGTGGAGTTTCATGTT |
| qPCR | IFNL1 | CTTCTTCCTCAGGGCTTCTT | GCTCAGTGCTCCAGATGTCT |
| qPCR | FAS | GGCTGTGGATCTGGGTGTTT | CAGTGTCTTGGAGGAGGAGC |
| qPCR | RPL30 | GCATGAGGCAACAACTGAAC | GCTTGCCTTTGTGGTTTGAT |
| qPCR | shRNA | CAGGGAGGTTGTTGCAAAAT | TGCCTCTCTGCTTGTTGTTC |
| FAIRE-qPCR and ChIP | FAS #1 | TGCCCATATACCATCCTCCTT | GGCTTGTCTCTGTTCCACCTT |
| FAIRE-qPCR and ChIP | FAS #2 | GAAAGCCCTCAGGAGGGTAA | AGCCCACTGTTCATCCTTCA |
| FAIRE-qPCR and ChIP | FAS #3 | GGACGATGCCAAAGGAATACT | TTCTTTCGTGCTCTGGTGAAC |
| FAIRE-qPCR and ChIP | FAS #4 | ATAGCTGGGGCTATGCGATT | GTTGTGGCTGCAACATGAGA |
| FAIRE-qPCR and ChIP | FAS #5 | GTGAGCCTCTCATGTTGCAG | GTTGGGGAGGTCTTGAAGGA |
| FAIRE-qPCR and ChIP | FAS #6 | CCCAGGTTGAACTACAGCAGA | GAAGTCAGGGTGAGGAAGGA |
| FAIRE-qPCR | IFNB1 #1 | TATTGGGCAAAACTCCTTGC | GGGAATTGAGCATCCTCTGA |
| FAIRE-qPCR | IFNB1 #2 | GATGTGCACTTGAAGTGGTG | TGGTCCGGGACGACGTGA |
| FAIRE-qPCR | IFNL1 #1 | CTTCTTCCTCAGGGCTTCTT | GCTCAGTGCTCCAGATGTCT |
| FAIRE-qPCR | IFNL1 #2 | TGATGGCATGCACCTGTAAT | CCTGGGTGATGGAGTGAGAT |
| FAIRE-qPCR | ATF3 | GGTTCTCCCGGGAAGCTATTAA | GAGAGCTGTGCAGTGCGCGCC |
| FAIRE-qPCR | ACTB | AAAGGCAACTTTCGGAACGG | TTCCTCAATCTCGCTCTCGC |
| FAIRE-qPCR | RPL30 | ACAGGGGTGGTCTATGTTGTC | CAGCACTTTCGGAGGTTGAGG |
| FAIRE-qPCR | shRNA #1 | CTGCAGGTCCGAGGTTCTAG | TCCCTATCAGTGATAGAGAAC |
| FAIRE-qPCR | shRNA #2 | GGTAGGCGTGTACGGTGG | GTCGCCACCATGAGCGAGC |
| FAIRE-qPCR | shRNA #3 | CTTCAAGTGCACATCCGAGG | CTCGTCCAGGCCGCGCAC |
| FAIRE-qPCR | shRNA #4 | TCGACATACGTTCTCTATCACT | TACACGCCTACCTCGACATAC |
| FAIRE-qPCR | shRNA #5 | CTTGATCAGCTCGCTCAT | GATGTCGAAGGCGAAGGGG |

**Supplementary Table S2. Antibodies used in this study**

| Target | Host | Source | Catalog no. | Dilution | Application |
|---|---|---|---|---|---|
| EFNB2 | Rabbit | Abcam | Ab131536 | 1:2000 | WB |
| ATF3 | Rabbit | Cell Signaling Technology | 18665 | 1:1000 | WB |
| p-eIF2α (Ser51) | Rabbit | Cell Signaling Technology | 3597 | 1:2000 | WB |
| eIF2α (total) | Rabbit | Cell Signaling Technology | 9722 | 1:2000 | WB |
| Cleaved Caspase-3 (Asp175) | Rabbit | Cell Signaling Technology | 9664 | 1:1000 | WB |
| Caspase-3 | Rabbit | Cell Signaling Technology | 9662 | 1:1000 | WB |
| β-Actin | Mouse | Sigma-Aldrich | A5441 | 1:5000 | WB |
| p-PKR (Thr451) | Rabbit | Cell Signaling Technology | 3075 | 1:2000 | WB |
| PKR | Rabbit | Cell Signaling Technology | 3072 | 1:2000 | WB |
| p-PERK (Thr980) | Rabbit | Cell Signaling Technology | 3179 | 1:1000 | WB |
| PERK | Rabbit | Cell Signaling Technology | 3179 | 1:2000 | WB |
| GCN2 | Mouse | R&D Systems | MAB6878 | 1ug/ml | WB |
| p-GCN2 (T899) | Rabbit | R&D Systems | AF7605 | 1:2000 | WB |
| FAS | Rabbit | Cell Signaling Technology | 4233 | 1:2000 | WB |
| Histone H3 | Rabbit | Cell Signaling Technology | 4499 | 1:2000 | ChIP |
| H3K9me3 | Rabbit | Cell Signaling Technology | 13969 | 1:1000 | ChIP |
| H3K27me3 | Rabbit | Cell Signaling Technology | 9733 | 1:1000 | ChIP |
| H3K4me3 | Rabbit | Cell Signaling Technology | 9751 | 1:1000 | ChIP |
| IgG (control) | Rabbit | Cell Signaling Technology | 2729 | 1:1000 | ChIP |
| Anti-rabbit IgG, HRP-linked | Goat | Cell Signaling Technology | 7074 | 1:5000 | WB |
| Anti-mouse IgG, HRP-linked | Goat | Cell Signaling Technology | 7076 | 1:5000 | WB |

## Supplementary Figure S1

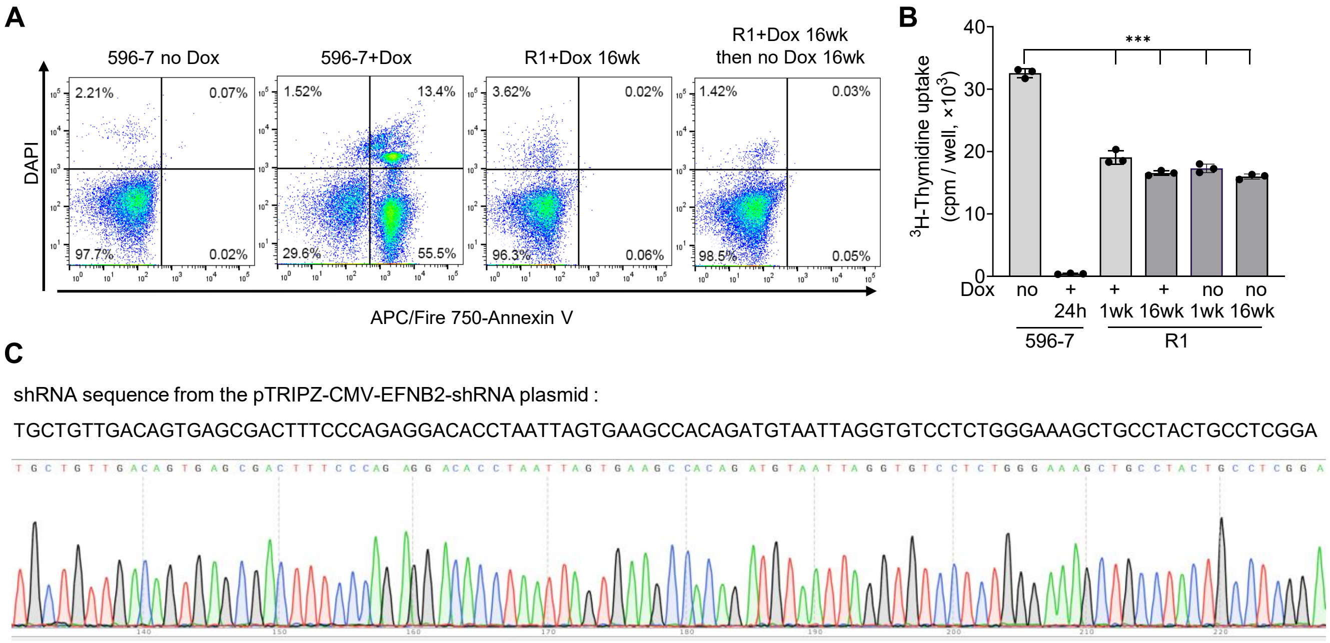


**Supplementary Figure S1. Cell death and proliferation in 596-7 and R1 cells. Integrity of the shRNA in R1 cells.**

A. Flow cytometry analysis of 596-7 and R1 cells stained with APC/Fire 750-Annexin V and DAPI. The 596-7 cells were cultured with or without Dox for 12 hours. R1 cells were cultured continuously with Dox for 16 weeks (wk) or first cultured continuously with Dox for 16wk and then deprived of Dox for 16 wk. Representative profiles from 3 experiments.

B. Cell proliferation by $^3$H-thymidine incorporation. 596-7 cells were cultured with or without Dox for 24 hours (h). R1 cells were cultured with Dox for 1 or 16 wk, or deprived of Dox for 1 or 16 wk, after being cultured continuously with Dox for 16 wk. The results reflect triplicate measurements; each dot represents one of the replicate

measurements; P values from unpaired Student's t-test are calculated relative to 596-7 no Dox; ***P<0.001. Representative of 3 experiments.

C. Sequence of the EFNB2-targeting shRNA (96 bp) used in this study. Results of shRNA sequencing from the pTRIPZ-CMV-EFNB2-shRNA plasmid confirm the integrity of the shRNA insert. No sequence mutation was detected.

# Supplementary Figure S2

**A**

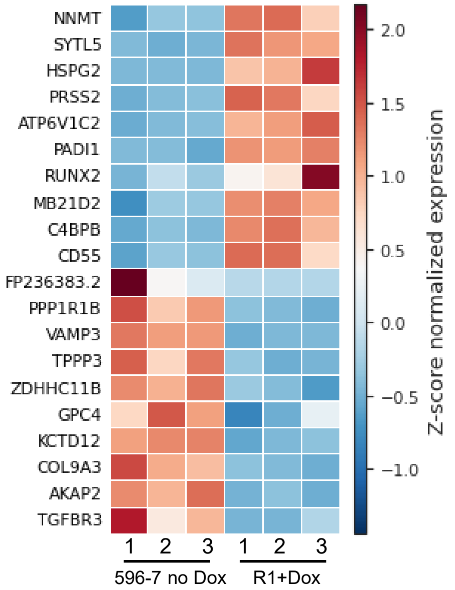


**B**

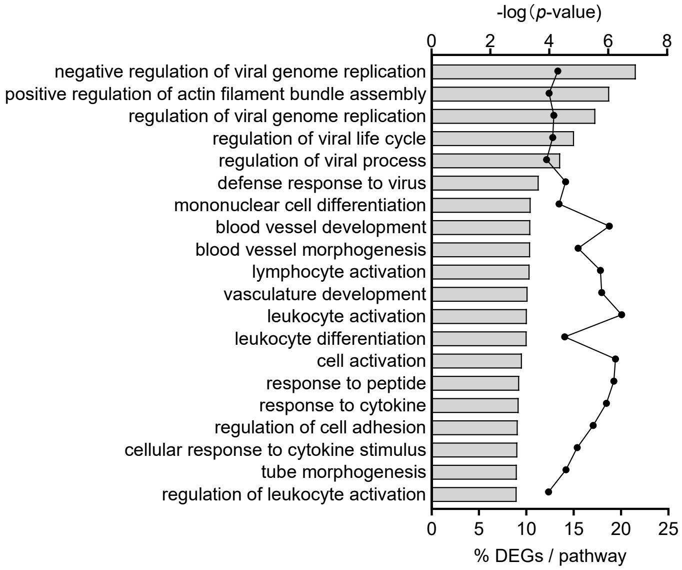


**C** Driver DNA mutations

| Gene | HT29 | 596-7 | R1 |
|---|---|---|---|
| APC | E853* | p.E853* | p.E853* |
| TP53 | R273H | p.R273H | p.R273H |
| BRAF | T119S | p.T119S | p.T119S |
| | V600E | p.V600E | p.V600E |
| PIK3CA | P449T | p.P449T | p.P449T |
| SMAD4 | Q311* | p.Q311* | p.Q311* |
| KRAS | Wild-type | Wild-type | Wild-type |

**D** Expression of mutant genes

| Gene | 596-7-1 | 596-7-2 | 596-7-3 | R1-1 | R1-2 | R1-3 | *P*-value |
|---|---|---|---|---|---|---|---|
| APC | 93.6 | 115.6 | 110.8 | 100.9 | 110.5 | 126.5 | 0.5827 |
| TP53 | 87.9 | 99 | 103.2 | 92.9 | 100.2 | 112.3 | 0.5213 |
| BRAF | 47.2 | 72 | 60.1 | 69.3 | 77.5 | 94.1 | 0.1154 |
| PIK3CA | 41.8 | 51.1 | 45.5 | 46.5 | 49.6 | 59.2 | 0.2975 |
| SMAD4 | 13.1 | 16.5 | 15.3 | 14.5 | 14.9 | 18.7 | 0.5432 |
| KRAS | 92.1 | 118.3 | 109.9 | 108.6 | 115.1 | 131.8 | 0.3193 |

**E** Known and new DNA mutations

| | 596-7 | R1 |
|---|---|---|
| Total identified mutations | 942 | 935 |
| | | |
| known mutations confirmed from Cosmic database | 277 | 281 |
| new mutations | 665 | 654 |
| among new mutations | | |
| splicing region mutations | 291 | 293 |
| CDS mutatioins | 374 | 361 |
| among new mutations | | |
| common mutations in 596-7/R1 | 595 | 595 |
| unique mutations in 596-7 | 70 | |
| unique mutations in R1 | | 59 |
| Total depth cut-off: minimum 10 | | |
| unique in R1 | | 18 |
| unique CDS mutations in R1 | | 11 |
| unique splicing mutations in R1 | | 7 |

**F** Unique DNA mutations detected in R1+Dox not in 596-7 no Dox

| | Gene Symbol | Variant_Type | HGVSc | Mutation Frequency (%) | Average expression |
|---|---|---|---|---|---|
| 1 | KLHL11 | DEL | c.36_38del | 21.4 | 48.67 |
| 2 | INTS2 | SNP | c.1481C>A | 18.5 | 32.68 |
| 3 | SIM2 | SNP | c.1138A>G | 18.2 | 13.21 |
| 4 | WNK2 | SNP | c.2858A>C | 23.1 | 16.22 |
| 5 | ZNF749 | SNP | c.1496T>A | 8.8 | 24.3 |
| 6 | DSCAM | SNP | c.229C>A | 11.5 | - |
| 7 | MAGEA10 | DEL | c.185_187del | 41.7 | - |
| 8 | IVL | INS | c.753_782dup | 12.5 | - |
| 9 | CAPN11 | SNP | c.1888C>A | 17.6 | - |
| 10 | CNTNAP3 | SNP | c.3640C>T | 14.3 | 0.33 |
| 11 | FAM230A | SNP | c.1171G>A | 12 | - |
| 12 | ATAD5 | Splice_Region | c.3137-7_3137-6del | 24.1 | 34.48 |
| 13 | ATAD5 | Splice_Region | c.3137-3_3137-2ins | 24.1 | 34.48 |
| 14 | LPO | Splice_Region | c.164+8_164+9ins | 30.4 | - |
| 15 | PAPOLG | Splice_Region | c.1769-4_1769-3ins | 12.5 | - |
| 16 | RABEP1 | Splice_Region | c.2216-7C>T | 23.5 | 85.31 |
| 17 | THADA | Splice_Region | c.2188-6_2188-4dup | 66.7 | 45.89 |
| 18 | DMP1 | Splice_Site | c.103-4_107del | 10.3 | - |

**Supplementary Figure S2. Transcriptomic and genomic analysis of R1 versus 596-7 cells.**

A. Top 10 and bottom 10 genes expressed at significantly different levels ($P<0.01$) in R1+Dox (R1 propagated with Dox) and uninduced 596-7 cells (never Dox) ($n=3$ replicates/group) displayed by row-wide Z score (color bar) listed in alphabetical order. Cut-off read average >5 (CPM-TMM counts). The numbers 1, 2 and 3 indicate RNA-seq samples.

B. Top 20 Gene Ontology (GO) Biological Processes showing differences between R1+Dox cells vs 596-7 (no Dox) cells. Differentially expressed genes (DEGs, $p<0.05$ and Fold Change>1.3) were applied to GO Biological Processes enrichment analysis. For each pathway, the bar represents the "% DEGs / pathway", calculated as the number of DEGs under the GO term divided by the total number of genes included in that GO term. The line-linked dots show the corresponding −log(*p*-value) derived from Fisher's exact test, reflecting the statistical significance of the enrichment. Higher "% DEGs / pathway" and larger −log(*p*-value) indicate stronger pathway enrichment.

C. Whole genome sequencing of R1 maintained with Dox and uninduced 596-7 cells reveals persistence of all known "driver" mutations reported in the parental HT29 cells line from the Catalog Of Somatic Mutations In Cancer (COSMIC).

D. Expression levels of the indicated mutant genes are similar in uninduced 596-7 and R1 cells maintained in Dox. RNA-seq analysis of triplicate samples shows no significant difference in expression levels of the indicated driver genes. P values (> 0.05 all comparisons) were calculated using unpaired Student's t-test.

E. Mutational differences between uninduced 596-7 and R1+Dox cells are limited to 18 somatic variants (with a sequencing depth cut-off of 10x). Analysis was performed by

MuTech2 software using the Genome Reference Consortium (GRCch38) human genome reference. Coding sequence (CDS) mutation: a change within gene CDS.

F. The 18 unique somatic mutations in R1+Dox cells, not detected in 596-7 cells, include 11 CDS mutations and 7 splicing mutations (splice region is indicated). HGVS (Human Gene Variation Society) nomenclature was used to describe variants. Gene symbols from Human Genome Organization (HUGO). Mutation frequency (%): mutation count/total depth of sequencing reads ranging from 8.8% to 66.7%; and average gene expression range 0 - 85.31 of R1 from RNA-seq (CPM-TMM counts).

**Supplementary Figure S3**

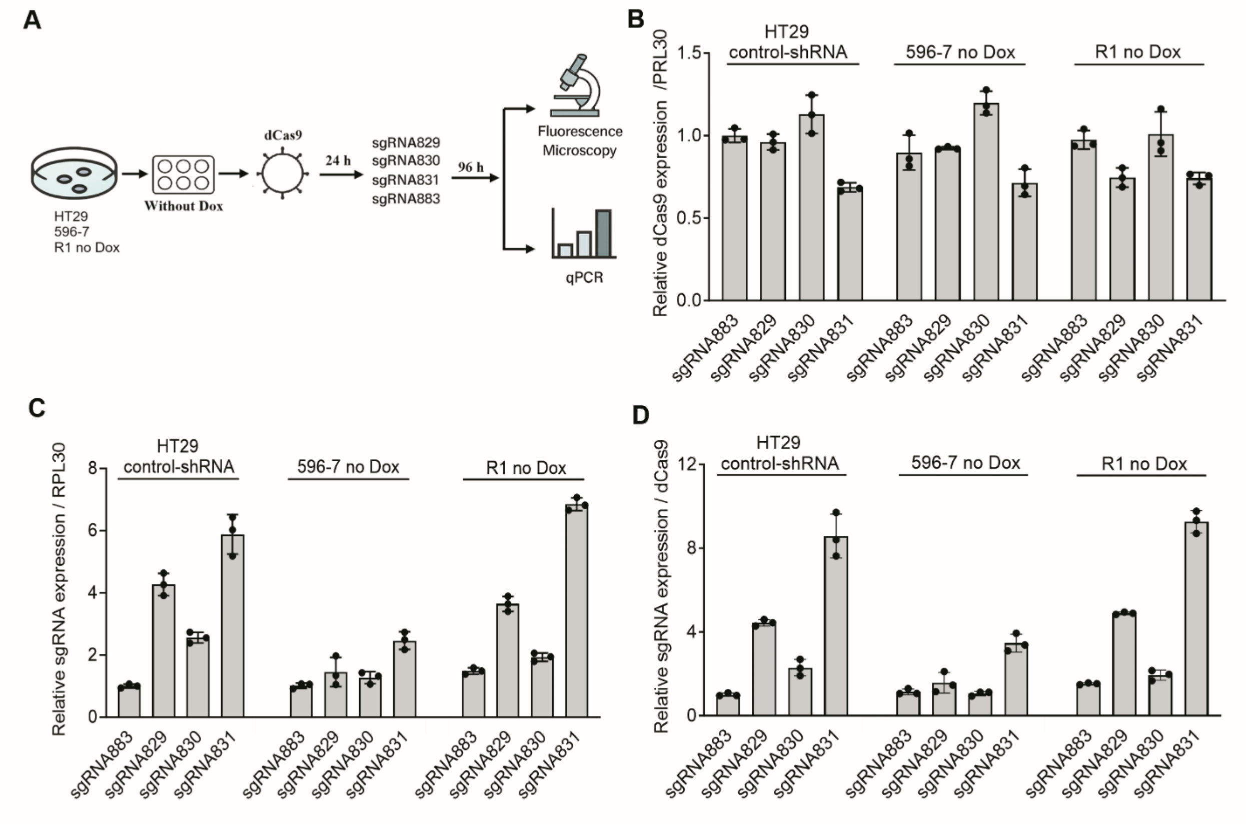


**Supplementary Figure S3. Expression of dCas9 and sgRNAs in HT29 and R1 cells.**

A. Schematic of the experiment testing reactivation of the endogenous shRNA by dCas9-sgRNA induction. HT29 control-shRNA (containing the non-Mammalian shRNA control SHC002), 596-7 cells (containing sh596), and R1 cells (derived from 596-7 cells and containing sh596) are infected with dCas9 lentivirus to induce expression of dCas9 protein. After 24 hours, cells are infected with the targeting sgRNAs (sgRNA829, sgRNA830, sgRNA831) or non-targeting control (sgRNA883). After 96 hours, RFP fluorescence is visualized and measured by qPCR. Levels of the endogenous shRNA (scrambled shRNA and sh596), dCas9 and sgRNAs were measured by qPCR.

B-D. Expression levels of dCas9 (B) and sgRNAs 883, 829,830 and 831 (C and D) in HT29 control-shRNA, 596-7 no Dox, and R1 no Dox cells. Expression of sgRNAs relative to RPL30 (C) and relative to dCas9 (D) in the indicated cell lines after infection with dCas9 virus followed by sgRNA. Results are presented as relative means (±SD), each dot represents one of the replicate measurements. Expression levels are normalized to HT29 control-shRNA infected with dCas9 and non-targeting sgRNA883. Representative of 2 experiments.

**Supplementary Figure S4**

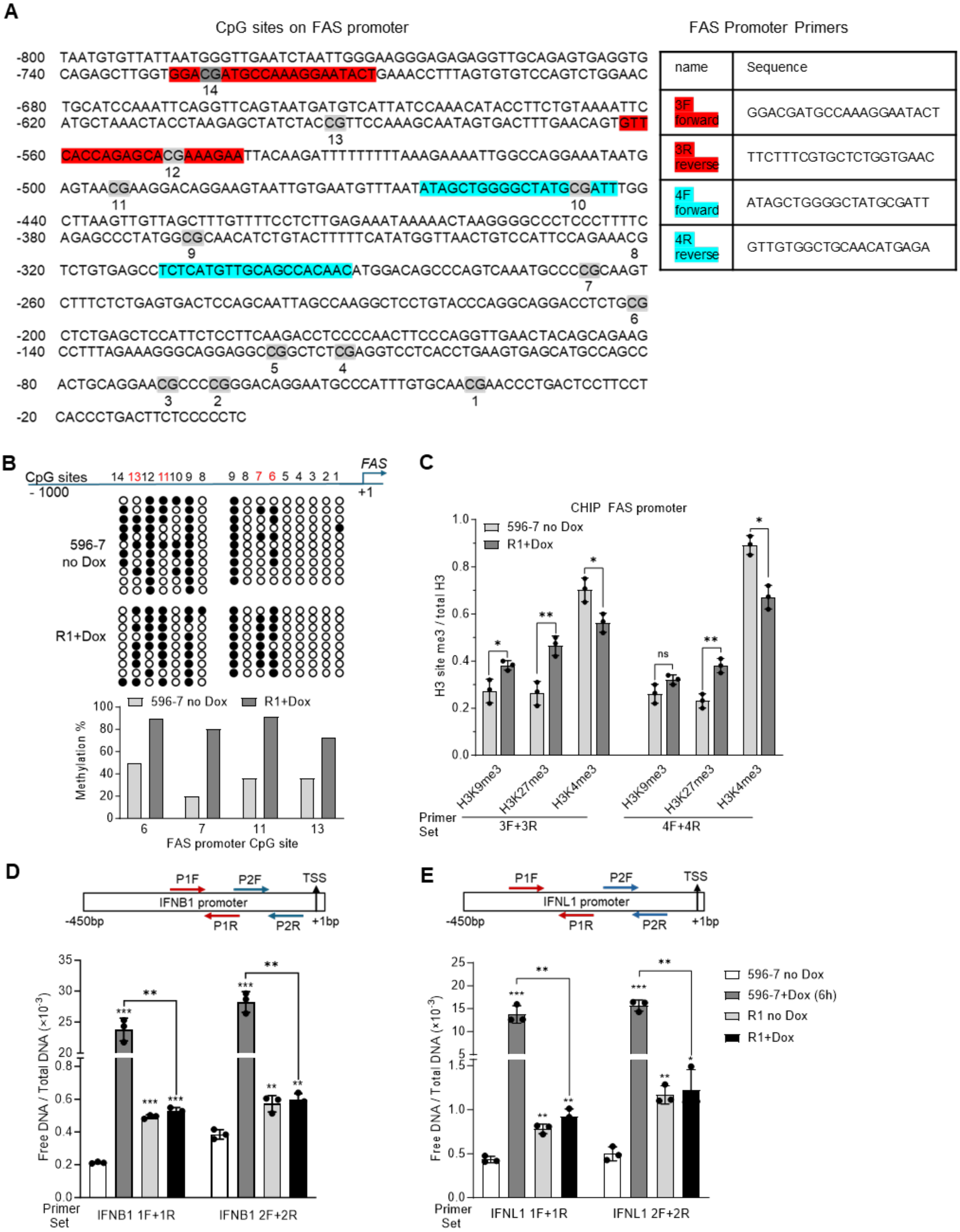

**Supplementary Figure S4. Methylation of CpG sites and histones in the *FAS* promoter and analysis of accessibility to chromatin in the *IFNB1* and *IFNL1* gene promoters.**

A. DNA sequence (800 bp) upstream of the *FAS* promoter transcription start-site (TSS). CpG sites are highlighted in grey, and location of primer pairs used for ChIP-qPCR below (panel C) are identified by red and teal highlights. Primer sets are shown on the right.

B. Methylation status of CpG sites identified by numbers (1-14) in the FAS promoter region of 597-7 cells never exposed to Dox and R1 cells maintained in Dox. The filled black circles identify methylation, and the open circles identify unmethylated CpG sites by bisulfite Sanger sequencing. The bar graph below shows the % methylation at CpG sites 6, 7, 11 and 13 in 596-7 and R1 cells.

C. Histone-3 (H3) trimethylation status (3me) at lysine-9 (H3K9me3), K27 (H3K27me3) and K4 (H3K4me3) in the FAS promoter region of 596-7 cells never exposed (596-7 no Dox) to Dox and R1 cells maintained in Dox (R1+Dox) by ChIP-qPCR analysis. The results are expressed as fold enrichment. The primers used are 3F+3R and 4F+4R as shown in panel A, right. Representative of 3 experiments.

D and E. Accessibility to chromatin in the *IFNB1* (D) and *IFNL1* (E) promoter regions 450 bp upstream of the TSS measured by FAIRE-qPCR in 596-7 exposed (6 hours 596-7+Dox) or never exposed to Dox (596-7 no Dox); R1 cells deprived of Dox for 4 months (R1 no Dox); and R1cells maintained in Dox for 4 months (R1+Dox). The results are presented as a ratio of free DNA/total input DNA (SD; triplicate measurements). Representative of 3 experiments.

In this figure, ns = not significant; *P<0.05, **P<0.01 and ***P < 0.001 by unpaired Student's t-test compared to 596-7 no Dox.

**Supplementary Figure S5**

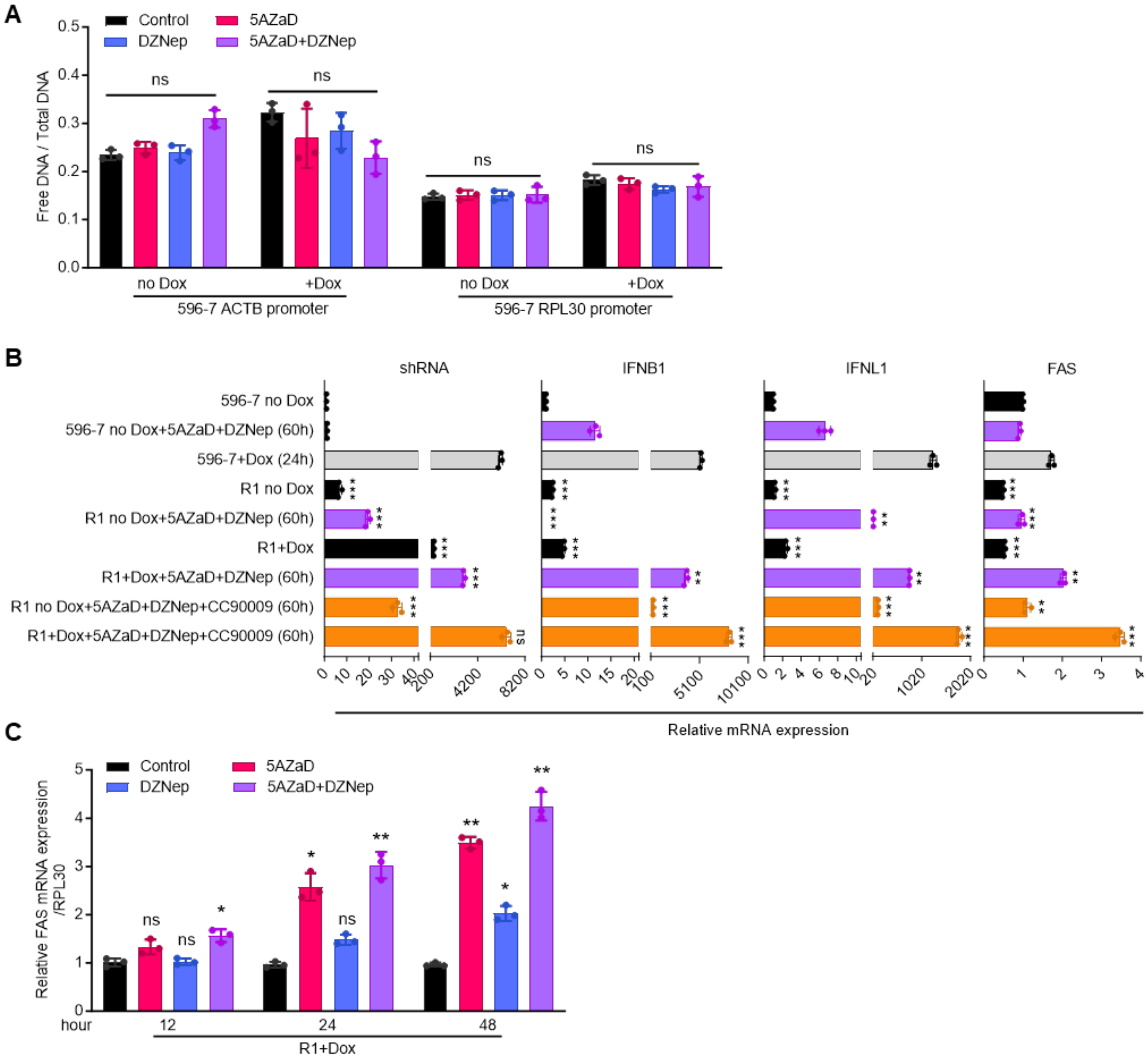


**Supplementary Figure S5. Dox effects on chromatin accessibility in 596-7 cells and R1 cells.**

A . FAIRE-qPCR analysis of chromatin accessibility at the *ACTB* and *RPL30* promoters in 596-7 cells cultured with (8 hours) or without Dox. The cells were tested after 60 hours incubation with medium only (includes 0.1% DMSO), 5AZaD (500 nM), DZNep (200

nM), or the combination of 5AZaD (500 nM) plus DZNep (200 nM). Error bars represent mean ± SD from triplicates; each dot represents one of the triplicates; representative of 3 experiments; P values from unpaired Student’s t-test are calculated relative to the untreated control; ns: not significant.

B. Relative mRNA expression of shRNA, *IFNB1*, *IFNL1* and *FAS* in the indicated cell populations cultured with or without Dox in medium only (includes 0.1% DMSO), 5AZaD (500 nM), DZNep (200 nM), the combination of 5AZaD (500 nM) plus DZNep (200 nM) or with the combination of 5AZaD (500 nM) plus DZNep (200 nM) plus CC-90009 (1 μM). Error bars represent mean ± SD from triplicates; each dot represents one of the triplicates; representative of 3 experiments; P values from unpaired Student’s t-test are calculated relative to 596-7+Dox (24 h); ns: not significant; *P<0.05, **P<0.01, ***P<0.001.

C. qPCR analysis of *FAS* mRNA expression in R1+Dox cells incubated 12 to 48 hours in medium only (includes 0.1% DMSO), with 5AZaD, DZNep, or the combination of 5AZaD + DZNep (as in A). Expression values, normalized by RPL30, are displayed as relative to expression in medium only. Error bars represent mean ± SD from triplicate measurements, each point represents one replicate. Representative of 3 experiments; P values from unpaired Student’s t-test are calculated relative to the untreated control; ns: not significant; *P<0.05, **P<0.01.

**Supplementary Figure S6**

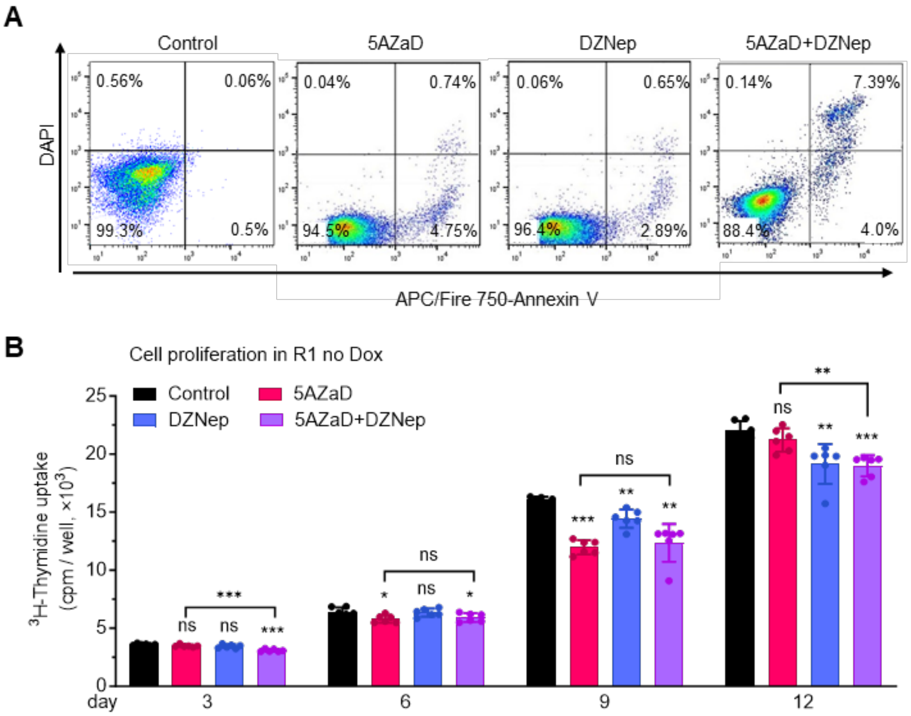


**Supplementary Figure S6. 5AZaD and DZNep reduce the growth and promotes cell death in R1 cells.**

A. Flow cytometry analysis of R1+Dox cells treated with 5AZaD (500 nM), DZNep (200 nM), or 5AZaD (500 nM) + DZNep (200 nM) for 3 days. APC/Fire 750-Annexin V and DAPI staining identifies cell death. Representative experiment of 3 performed.

B. Cell proliferation measured by $^{3}$H-thymidine incorporation in R1 cells propagated without Dox for 4 months; cells were incubated with 5AZaD (500 nM), DZNep (200 nM), or 5AZaD (500 nM) + DZNep (200 nM) for 3 to 12 days. Data represent the mean (± SD) from six replicates; representative experiment of 4 performed; each point represents one the replicates; P values from unpaired Student's t-test are calculated relative to the untreated control; ns: not significant; *P<0.05, **P<0.01, ***P<0.001.

**Supplementary Figure S7**

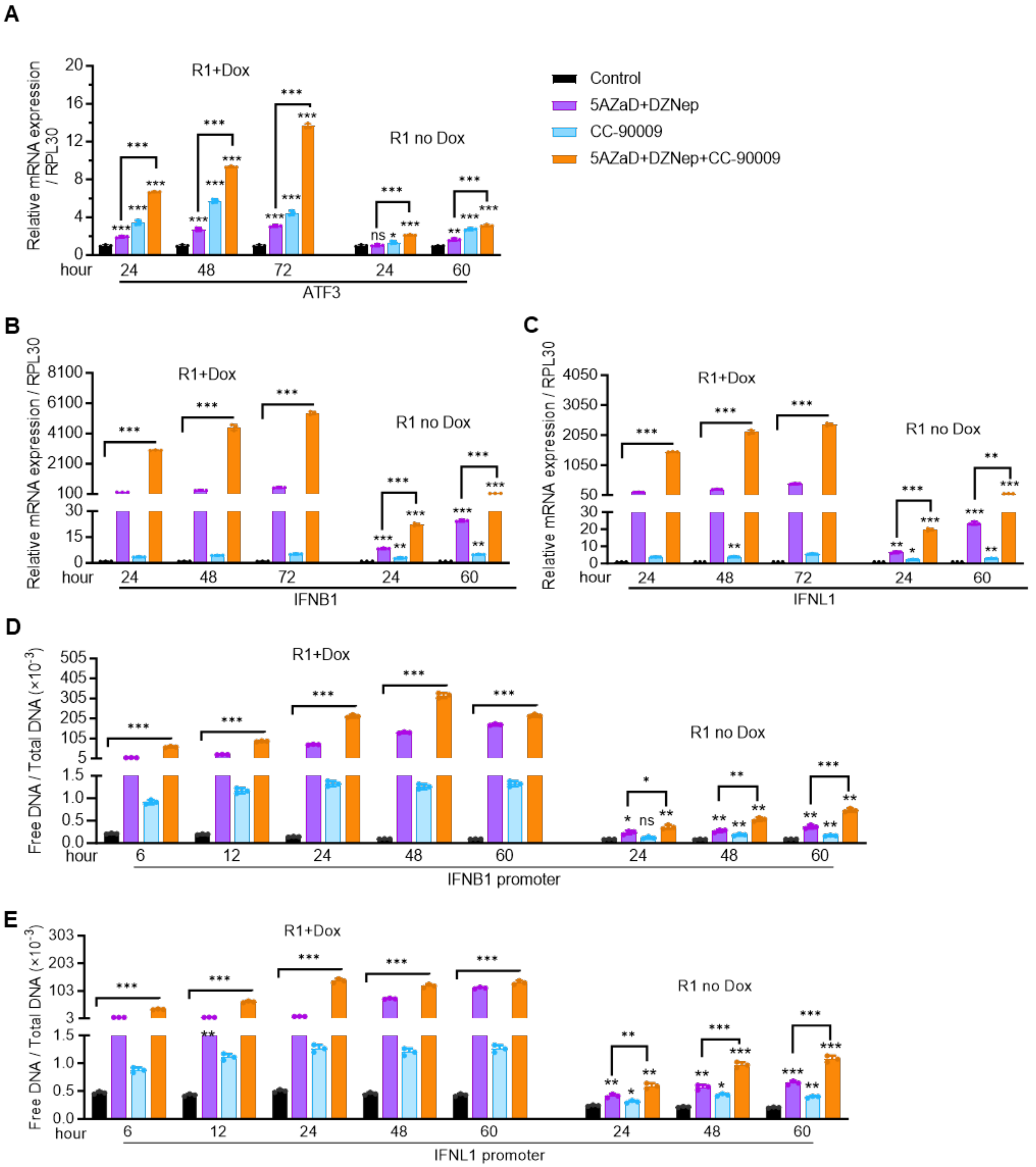

**Supplementary Figure S7. Effects of 5AZaD, DZNep and CC-90009 on expression of ISR-related genes *ATF3*, *IFNB1* and *IFNL1* and chromatin accessibility in R1 cells propagated with or without Dox.**

A-C. Relative *ATF3* (A), *IFNB1* (B), and *IFNL1* (C) expression in R1 cells, propagated in Dox (R1+Dox) or without Dox (4 months; R1 no Dox), incubated in medium only (with 0.1% DMSO), with 5AZaD+DZNep, CC-90009, or 5AZaD+DZNep+CC-90009 for the indicated number of hours. Results from qPCR, normalized by RPL30, are displayed as relative to expression in medium (with 0.1% DMSO).

D and E. FAIRE-qPCR analysis of accessibility to chromatin at the *IFNB1* (D) and *IFNL1* (E) promoters in R1 cells, propagated in Dox (R1+Dox) or without Dox (4 months; R1 no Dox), incubated in medium only (with 0.1% DMSO), with 5AZaD+DZNep, CC-90009, or 5AZaD+DZNep+CC-90009 for the indicated number of hours.

In this figure, 5AZaD (500 nM), DZNep (200 nM), CC-90009 (1 μM); error bars represent the mean (± SD) from triplicate measurements, each point represents one replicate. All panels in this figure are representative of 3 experiments. P values from unpaired Student's t-test are calculated relative to the untreated control; ns: not significant; *P<0.05, **P<0.01, ***P<0.001.

**Supplementary Figure S8**

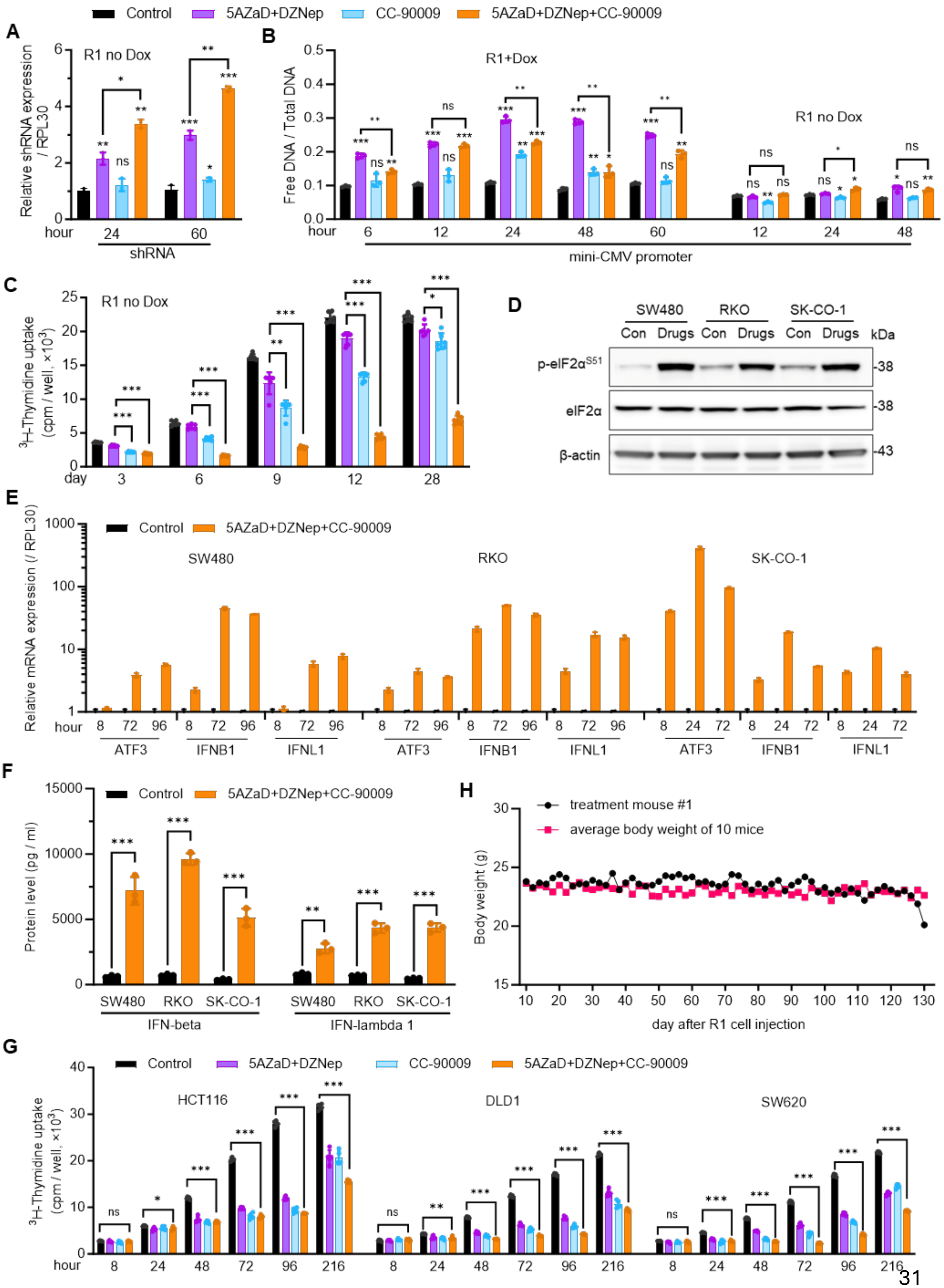

**Supplementary Figure S8. Effects of the combined drugs 5AZaD, DZNep and CC-90009 on gene and shRNA expression, chromatin accessibility, ISR and cell proliferation in R1 and other colon carcinoma cells**

A. Relative shRNA expression in R1 cells maintained without Dox (4 months) incubated in medium only (with 0.1% DMSO, R1 no Dox), with 5AZaD+DZNep, CC-90009, or 5AZaD+DZNep+CC-90009 for 24 or 60 hours. Results from qPCR, normalized by RPL30, are displayed as relative to expression in medium (with 0.1% DMSO).

B. FAIRE-qPCR analysis of accessibility to chromatin at the shRNA mini-CMV promoter in R1 cells maintained with Dox (R1+Dox) or without Dox (4 months, R1 no Dox). R1 cells with or without Dox were incubated in culture medium only (with 0.1% DMSO) or with 5AZaD+ DZNep, CC-90009, or 5AZaD+DZNep+CC-90009 for the indicated number of hours.

C. Proliferation of R1 cells cultured without Dox (4 months) in medium only (with 0.1% DMSO), with 5AZaD+DZNep, CC-90009, or 5AZaD+DZNep+CC-90009 for the indicated number of days. Proliferation was measured by $^{3}$H-thymidine incorporation. Data represent means (± SD) from six cultures.

D. Activation of the ISR in SW480, RKO and SK-CO-1 cells cultured in medium only (with 0.1% DMSO; Con) or with 5AZaD+DZNep+CC-90009; for 24 (SK-CO-1) or 72 (SW480 and RKO) hours.

E. Relative expression of *ATF3*, *IFNB1*, and *IFNL1* in the colon carcinoma cell lines SW480, RKO and SK-CO-1 cultured in medium only (with 0.1% DMSO) or with 5AZaD+DZNep+CC-90009 for the indicated number of hours. Results from qPCR are normalized by RPL30.

F. Protein levels of IFN-beta and IFN-lambda1 in the colon cancer cell lines SW480, RKO and SK-CO-1 cultured in medium only (with 0.1% DMSO) or with 5AZaD+DZNep+CC-90009 for 24 (SK-CO-1) or 72 (SW480 and RKO) hours.

G. Proliferation of HCT116, DLD1, and SW620 colorectal cancer cells incubated in medium only (with 0.1% DMSO), with 5AZaD+DZNep, CC-90009, or with 5AZaD+DZNep+CC-90009 for the indicated number of hours. Data represent means ± SD from six replicate cultures.

H. Body weight monitoring of R1+Dox xenografts in NOD/SCID mice treated with the triple combination of 5AZaD (1 mg/kg) + DZNep (0.5 mg/kg) + CC-90009 (2.5 mg/kg) mice (n=10). Red line means the average of 10 mice; the black line shows the body weight of mouse #1, which died on day 130.

In the in vitro experiment of this figure, 5AZaD (500 nM), DZNep (200 nM), CC-90009 (1 µM); error bars represent the means ± SD from three or six measurements, each point represents one replicate. Experiments shown in A, B, C, F and G are representative of 3 experiments. P values from unpaired Student's t-test are calculated relative to the untreated control; ns, not significant; *P<0.05, **P<0.01, ***P<0.001.
