# Supplementary material for: Targeting the epigenome and the integrated stress response to normalize colorectal cancer subclonal plasticity and progression
Source: Cell Death Dis. 2026 Apr 10;17(1):459. doi: 10.1038/s41419-026-08720-2 (PMC13181133; doi:10.1038/s41419-026-08720-2)
Supplement: Supplementary file 2 — Supplemental Tables 1 and 2 in editable format [file 41419_2026_8720_MOESM2_ESM.pdf]

Figure 1 Panel G

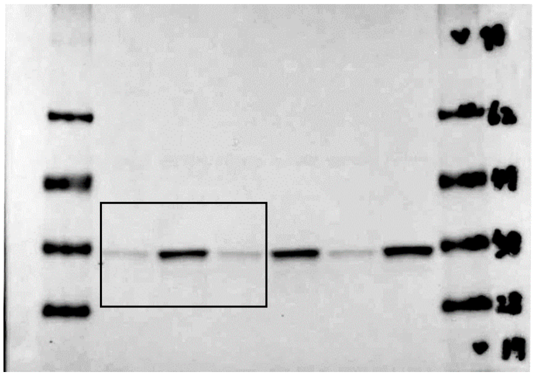

p-eIF2α^{S51}

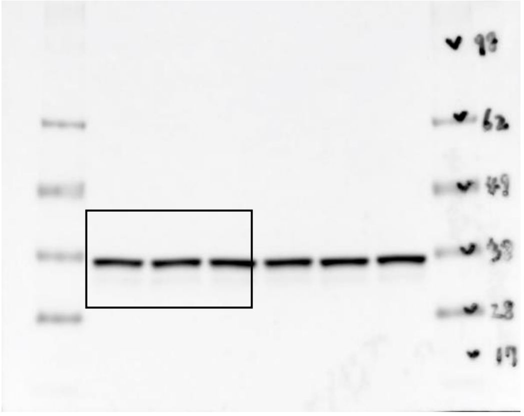

eIF2α

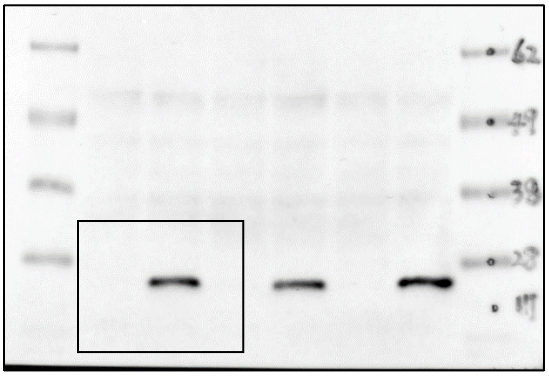

ATF3

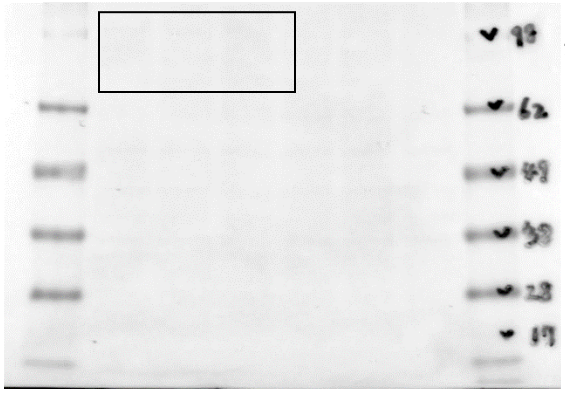

p-PERK^{T980}

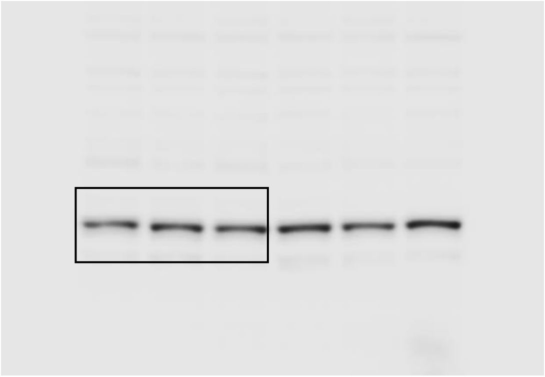

β-actin

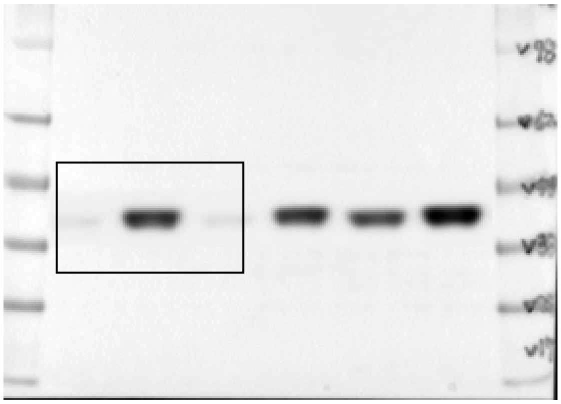

ATF4

62kDa

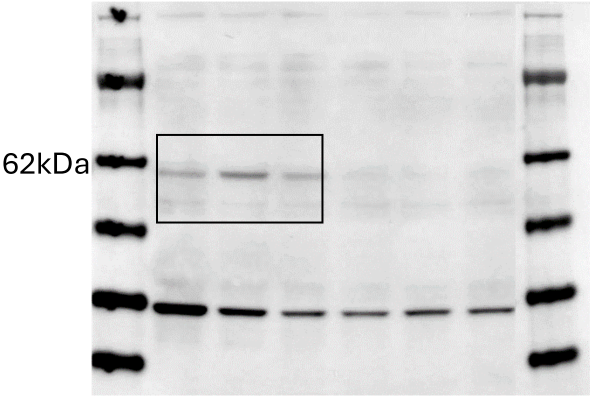

p-PKR^{T451}

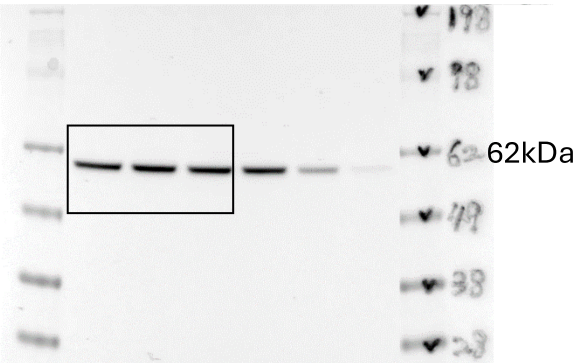

62kDa

PKR

198kDa

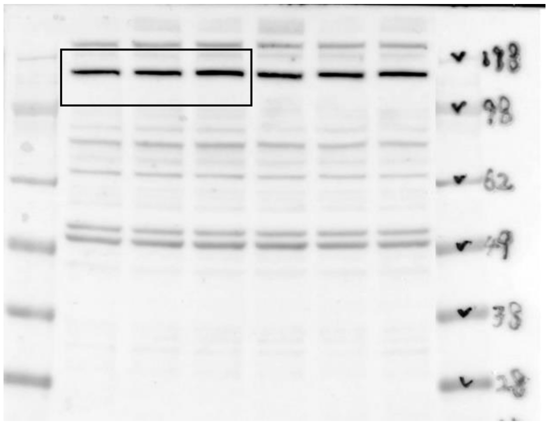

GCN2

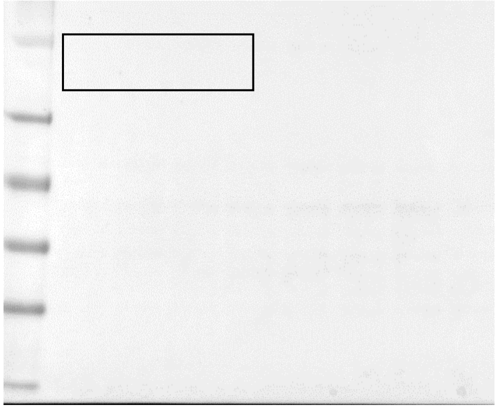

198kDa

p-GCN2^{T899}

Figure 4

Panel D

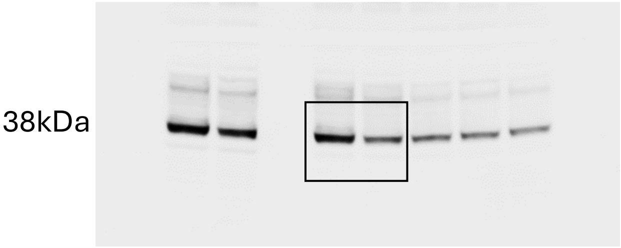

FAS

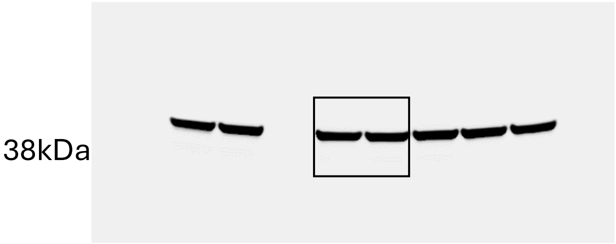

β-actin

Figure 5

Panel G

38kDa

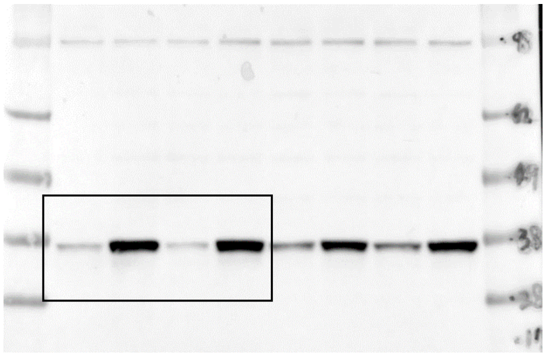

p-eIF2α^{S51}

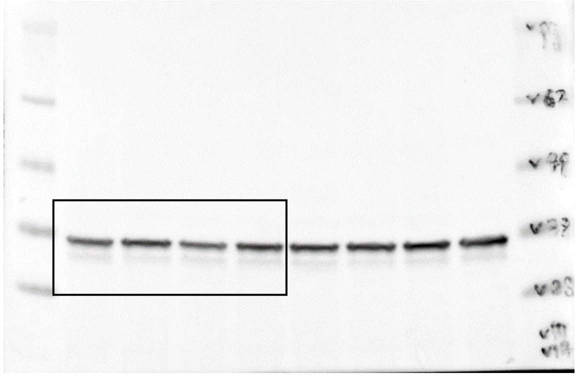

eIF2α

38kDa

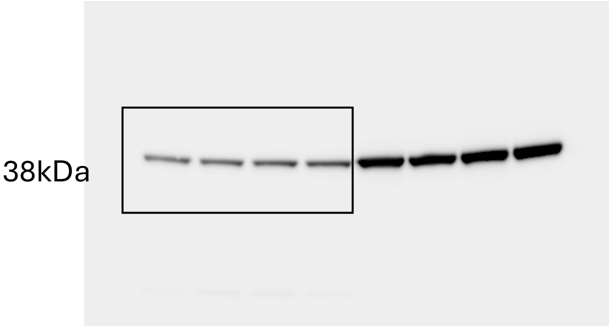

β-actin

28kDa

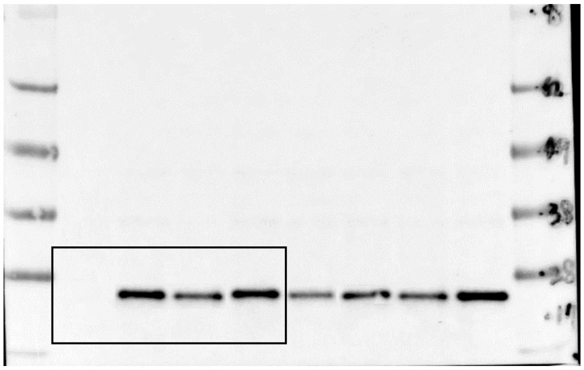

ATF3

28kDa

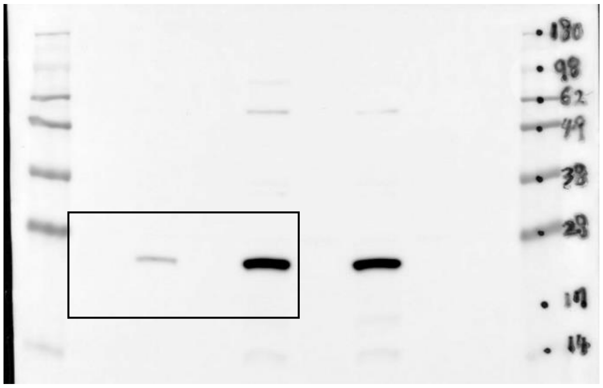

Cleaved caspase-3

Figure 7

Panel A

38kDa

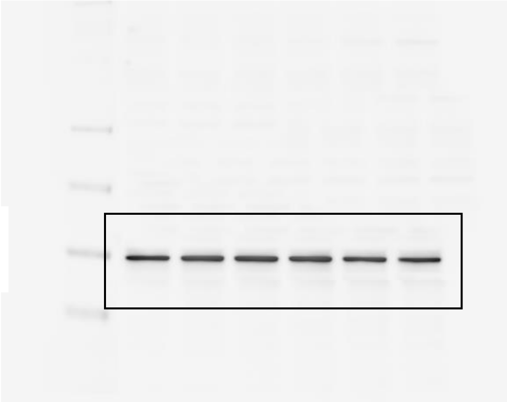

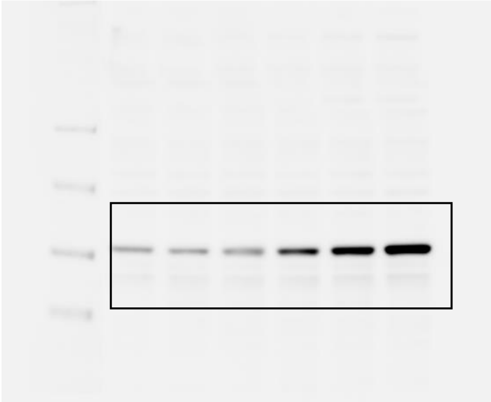

38kDa

24h eIF2α

24h p-eIF2α^{S51}

38kDa

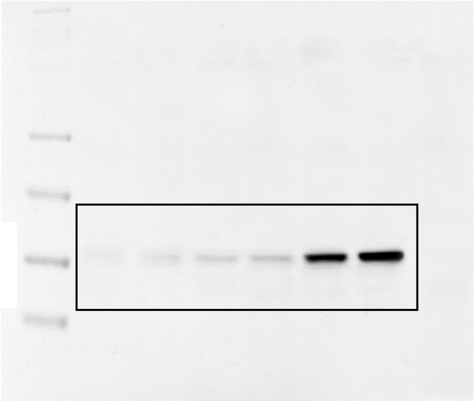

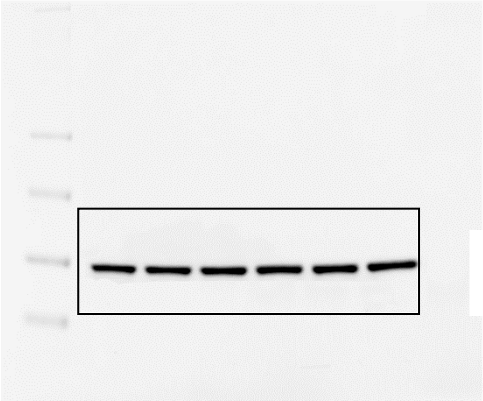

38kDa

24h eIF2α

24h p-eIF2α^{S51}

38kDa

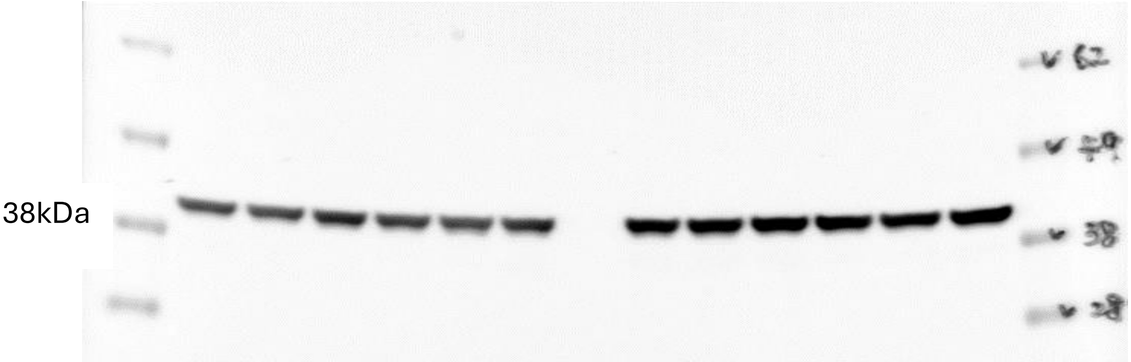

24h β-actin

8h β-actin
